# Activation by O_2_ of Ag_*x*_Pd_1–*x*_ Alloy Catalysts for Ethylene Hydrogenation

**DOI:** 10.1021/acscatal.3c03253

**Published:** 2023-10-28

**Authors:** Nicholas Golio, Andrew J. Gellman

**Affiliations:** ^†^Department of Chemical Engineering ^‡^W.E. Scott Institute for Energy Innovation, Carnegie Mellon University 5000 Forbes Ave., Pittsburgh, Pennsylvania 15213, United States

**Keywords:** catalysis, palladium, silver, oxygen, ethylene hydrogenation, thin films, high-throughput

## Abstract

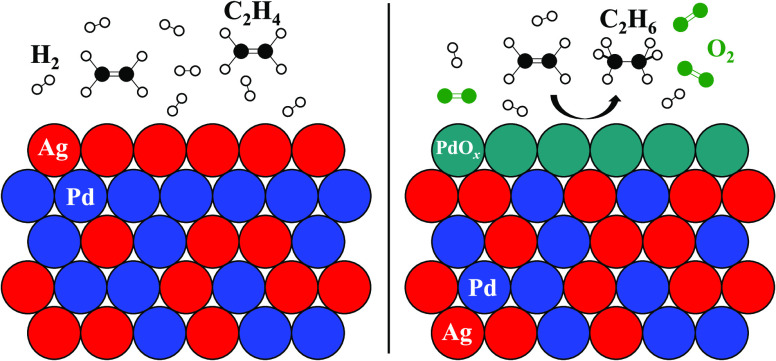

A composition spread alloy film (CSAF) spanning all of Ag_*x*_Pd_1–*x*_ composition space, *x*_Pd_ = 0 → 1, was used to study catalytic ethylene hydrogenation with and without the presence of O_2_ in the feed gas. High-throughput measurements of the ethylene hydrogenation activity of Ag_*x*_Pd_1–*x*_ alloys were performed at 100 Pd compositions spanning *x*_Pd_ = 0 → 1. The extent of ethylene hydrogenation was measured versus *x*_Pd_ at reaction temperatures spanning *T* = 300 → 405 K and inlet hydrogen partial pressures spanning *P*_H2_^in^ = 70 → 690 Torr. The inlet ethylene partial pressure was constant at *P*_C2H4_^in^ = 25 Torr, and the O_2_ inlet partial pressure was either *P*_O2_^in^ = 0 or 15 Torr. When *P*_O2_^in^ = 0 Torr, only those alloys with *x*_Pd_ ≥ 0.90 displayed observable ethylene hydrogenation activity. As expected, the most active catalyst was pure Pd, which yielded a maximum conversion of ∼0.4 at *T* = 405 K and *P*_H2_^in^ = 690 Torr. Adding a constant O_2_ partial pressure of *P*_O2_^in^ = 15 Torr to the feed stream dramatically increased the catalytic activity across the CSAF at all experimental conditions and catalyst compositions without inducing catalytic ethylene combustion and without measurable O_2_ consumption. The presence of *P*_O2_^in^ = 15 Torr more than doubled the maximum achievable conversion on Pd to ∼0.9 and activated alloys with as little as *x*_Pd_ = 0.6 for ethylene hydrogenation. Measurement of the reaction order with respect to hydrogen, *n*_H2_, showed that *n*_H2_ ≈ 0 when *P*_O2_^in^ = 15 Torr on high *x*_Pd_ alloys but that *n*_H2_ increases to values between 0.5 and 1 as *x*_Pd_ decreases or when *P*_O2_^in^ = 0 Torr. We attribute this *P*_O2_^in^-induced change in *n*_H2_ to a change in the reaction mechanism resulting from different functional catalyst surfaces: one that is O_2_-activated and Pd-rich and one that is Ag-capped with low activity. Both are extremely sensitive to the bulk alloy composition, *x*_Pd_, and the reaction temperature, *T*. These results show that the activity of AgPd catalysts for ethylene hydrogenation depends strongly on the operational conditions. Furthermore, we demonstrate that the exposure of AgPd catalysts to 15 Torr of O_2_ at moderate temperatures leads to enhanced catalyst performance, presumably by stimulating both Pd segregation to the topmost surface and Pd activation for ethylene hydrogenation.

## 1 Introduction

Catalytic hydrogenation reactions are used for a wide range of industrial applications, such as the production of fuels, pharmaceuticals, and even for waste management.^1−4^ Since its discovery at the end of the 19th century, alkene hydrogenation over heterogeneous catalysts has been studied extensively in the literature.^5−8^ Ethylene (C_2_H_4_) is a model reactant for these systems since it is the simplest hydrocarbon containing a C=C bond, making it an obvious candidate for probing the hydrogenation of larger, more complex olefins.^9^ In this work, ethylene hydrogenation is investigated on Ag_*x*_Pd_1–*x*_ binary alloy catalysts with compositions spanning the entire range *x*_Pd_ = 0 → 1. The catalysts have been fabricated in the form of high-throughput libraries referred to as composition spread alloy films (CSAFs).^10,11^ Measurements of catalytic activity have been made on the AgPd CSAF using a multichannel microreactor array of our own design.^12^

Ethylene hydrogenation to ethane (C_2_H_6_) on Pd is easily achieved at ambient temperature and pressure and has been shown to produce no byproducts.^13^ Alloys that contain Ag are of particular interest because they are capable of catalyzing highly selective reactions due to their relatively weak binding of reaction intermediates.^4,14−17^ Thus, probing catalysts within AgPd composition space will assist in the discovery of highly active and selective catalysts for the partial hydrogenation of alkynes^16,18−21^ and acrolein.^22^

The inherently low hydrogenation activity of Ag is demonstrated by the absence of measurable activity for the hydrogenation of many molecules, including alkenes,^23−27^ alkynes,^28,29^ aldehydes,^30,31^ and alcohols,^17,32,33^ on its clean, low Miller index surfaces. Furthermore, H_2_ does not dissociate on clean Ag surfaces, and atomic H is only very weakly bound, desorbing from the surface at temperatures below room temperature in temperature-programmed desorption (TPD) experiments.^14,34,35^ By contrast, it is known that H_2_ has a negligible barrier to dissociation on Pd.^36−39^ Since Pd has a much lower barrier to H_2_ dissociation than Ag, it is often the more useful catalyst for hydrogenation reactions. However, the weak binding of H to Ag can be advantageous in AgPd catalysts for reactions in which a partially hydrogenated product is desired. The potential for identifying highly selective reactions on relatively inert Ag is a catalyst design challenge that can be realized through the coadsorption of H atoms and reactant molecules with C=C and/or C=O bonds. Binary AgPd alloys provide a promising composition search space for catalysts that possess both high activity and high selectivity for reactions such as olefin, aldehyde, and ketone hydrogenation.

The key to creating AgPd alloy catalysts that retain the selectivity of Ag without compromising their reactivity is to ensure that the more active Pd atoms are on or near the topmost surface. The location and distribution of Pd on the surface strongly affect the overall performance of the catalyst. Ensuring that Pd is present on the surface to promote reactivity is complicated by the fact that Ag has a substantially lower surface free energy than Pd.^40^ Ab initio calculations^40^ have found that the surface energy of FCC(111) slabs of Ag and Pd are γ_Ag_ = 1.2 J/m^2^ and γ_Pd_ = 1.6 J/m^2^, respectively, and experimental measurements^41^ have found that γ_Pd_ can be as high as 2.1 J/m^2^. Since the surface energy of Ag is at least 25% lower than that of Pd, clean surfaces of AgPd alloys are expected to be highly enriched in surface-segregated Ag.^42−44^ However, adsorbates can induce the resegregation of Pd to the top surface if they are more strongly bonded to Pd than to Ag and if the temperature allows sufficient mobility of Pd in Ag. Recently, density functional theory (DFT)-based Monte Carlo simulations have shown resegregation of Pd to the surface when AgPd alloys are exposed to acetylene.^45,46^ A study incorporating both modeling and experiment shows that Pd in the immediate subsurface of a Ag(111) crystal segregates to the surface in the presence of adsorbed CO or O_2._^47^

In this work, ethylene hydrogenation kinetics on a Ag_*x*_Pd_1–*x*_ composition spread alloy film (CSAF) were measured with and without O_2_ in the feed to elucidate their dependence on Ag_*x*_Pd_1–*x*_ alloy composition. These measurements support the hypothesis that O_2_ can activate a Ag_*x*_Pd_1–*x*_ catalyst by inducing Pd segregation without resulting in catalytic ethylene combustion. A relevant study^48^ of ethylene combustion on a Pd(100) single crystal reported no measurable combustion products at reaction temperatures below 428 K, even for O_2_/ethylene ratios as high as 3, which far exceed the conditions used for our experiments. In this work, ethylene hydrogenation was performed at reaction temperatures from *T* = 300 → 405 K, and the O_2_/ethylene ratio was fixed at either 0 or 0.6. The catalysts were exposed to hydrogen partial pressures from *P*_H2_^in^ = 70 → 690 Torr with the inlet ethylene pressure held constant at *P*_C2H4_^in^ = 25 Torr and the inlet O_2_ pressure either *P*_O2_^in^ = 0 or 15 Torr. When the reaction was performed in the absence of O_2_, only those alloys with *x*_Pd_ ≥ 0.9 displayed measurable activity for ethylene hydrogenation at *T* ≤ 405 K. In contrast, the presence of *P*_O2_^in^ = 15 Torr at otherwise identical conditions lead to a significant increase in the ethylene hydrogenation activity across the film, including the activation of alloys with bulk Pd contents as low as *x*_Pd_ = 0.6. This occurs without any observable consumption of O_2_, i.e., no evidence of either ethylene combustion or H_2_ combustion. We attribute this increase in the ethylene hydrogenation activity to the ability of O_2_ to facilitate the segregation and activation of Pd atoms on the top surface of the alloy, thus increasing the number of active sites available for ethylene hydrogenation. This work supports the conclusion drawn from others that the performance of catalytic materials can be profoundly changed by their operational conditions. In our case, the interaction of O_2_ with AgPd alloys and the proposed change to the catalyst is directly observed in the ethylene hydrogenation activity of the Ag_*x*_Pd_1–*x*_ CSAF when O_2_ is present in the feed.

## 2 Experimental Section

The rates of ethylene hydrogenation were measured at 100 compositions of Ag_*x*_Pd_1–*x*_ spanning the range *x*_Pd_ = 0 → 1. This was accomplished using a AgPd CSAF and a multichannel microreactor array capable of isolating 100 regions of the CSAF, each having a different alloy composition.^11,12^

### 2.1 CSAF Preparation

The CSAF was prepared by physical vapor deposition of Ag and Pd onto a 14 × 14 × 3 mm^3^ polished Mo substrate (Valley Design Corp.) using a rotatable shadow mask CSAF deposition tool that has been described previously.^10^ Mo was chosen as the substrate material because it does not alloy with Ag or Pd during the deposition or at the reaction temperatures.^49−52^ The deposition rates from the Ag and Pd electron beam evaporation sources were controlled independently by their heating power sources and were calibrated using a quartz crystal microbalance (QCM). The film thickness was controlled by the deposition time and reached a uniform thickness of ∼100 nm. The orientation of the shadow masks 180° from one another resulted in opposing flux gradients of Ag and Pd across the substrate. The CSAF was deposited and then annealed at 800 K for 1 h in ultrahigh vacuum (UHV) conditions. These conditions are sufficient to induce film crystallization.^50,53^

### 2.2 Characterization of CSAF Composition

Energy-dispersive X-ray spectroscopy (EDX) of the Ag_*x*_Pd_1–*x*_ CSAF was performed in a Tescan VEGA3 scanning electron microscope (SEM) to map the bulk alloy composition across the substrate and measure the overall film thickness. The CSAF was positioned by an automated stage, allowing analysis across a grid of 13 × 13 evenly spaced measurement points spanning the 12 × 12 mm^2^ area at the center of the substrate. The electron beam energy was set to 20 keV, and the EDX scan area of each point was 50 × 50 μm^2^. At each measurement point, a scan from 0–10 keV was performed since it contained all of the characteristic X-ray energies emitted from Ag, Pd, and Mo.^54^ The bulk alloy composition corresponding to each spectrum was quantified using the Oxford Instruments INCA ThinFilmID software package, which accounted for the morphology of a thin AgPd film deposited on a Mo substrate. The overall film thickness at each point was calculated by comparing the Ag and Pd signal intensities at each point to those of a Ni reference material.

### 2.3 Measurement of Ethylene Hydrogenation Activity

The ethylene hydrogenation activity of the Ag_*x*_Pd_1–*x*_ CSAF was measured at 100 different alloy compositions using a high-throughput multichannel microreactor array, which has been described in detail elsewhere.^12^ Reactant mixtures of H_2_, C_2_H_4_, Ar, and O_2_ were delivered continuously to 100 isolated regions of the Ag_*x*_Pd_1–*x*_ CSAF surface, and the products were continuously withdrawn from each region for analysis using a Stanford Research Systems quadrupole mass spectrometer (RGA-200).

The ethylene hydrogenation activity of the Ag_*x*_Pd_1–*x*_ catalysts contained on the CSAF was measured at atmospheric pressure (*P*^tot^ = 760 Torr) and over a temperature range from *T* = 300 → 405 K in increments of 15 K. Two principle sets of flow conditions were used, one with 15 Torr of O_2_ in the feed and one with no O_2_ present. In both cases, the H_2_ inlet partial pressure spanned the range *P*_H2_^in^ = 70 → 690 Torr, and the ethylene inlet partial pressure was constant at *P*_C2H4_^in^ = 25 Torr with Ar constituting the remainder of gas flow. The combined total flow rate of 10 mL/min was split equally between the 100 microreactor channels and two reference channels. The reference channels have 0% ethylene conversion and 100% ethylene conversion, respectively. The reference channel with 0% conversion delivered the reactant gas mixture directly to the gas sampling system. The reference channel with 100% conversion is a coiled stainless-steel tube (W.W. Grainger, Inc., 0.02″ ID, 0.028″ OD) loaded with ∼60 cm of Pd wire (ESPI Metals, Diameter: 0.004″, Purity: 3N5) that was independently heated using a heating mantle (Glas-Col). Mass flow controllers (Aalborg GFC-17) were used to regulate the flow rates of H_2_ (99.999%, Valley National Gases), C_2_H_4_ (99.995%, Valley National Gases), Ar (99.999%, Valley National Gases), and O_2_ (99.98%, Valley National Gases) through the microreactor system. The exposed surface area of the catalysts on the CSAF is defined by the 700 × 800 μm^2^ holes in the elastomer gasket (Kalrez 7075, Dupont) that forms an airtight seal between the microreactor channels and the catalyst film. Experiments were performed by keeping the reaction temperature constant and varying the H_2_ inlet partial pressure, allowing the system to reach a steady state by waiting 1.5 h after each change of flow conditions. Once the system was at a steady state, three sequential scans through all 100 outlet channels were performed to measure the catalytic activity.

**Figure 1:**
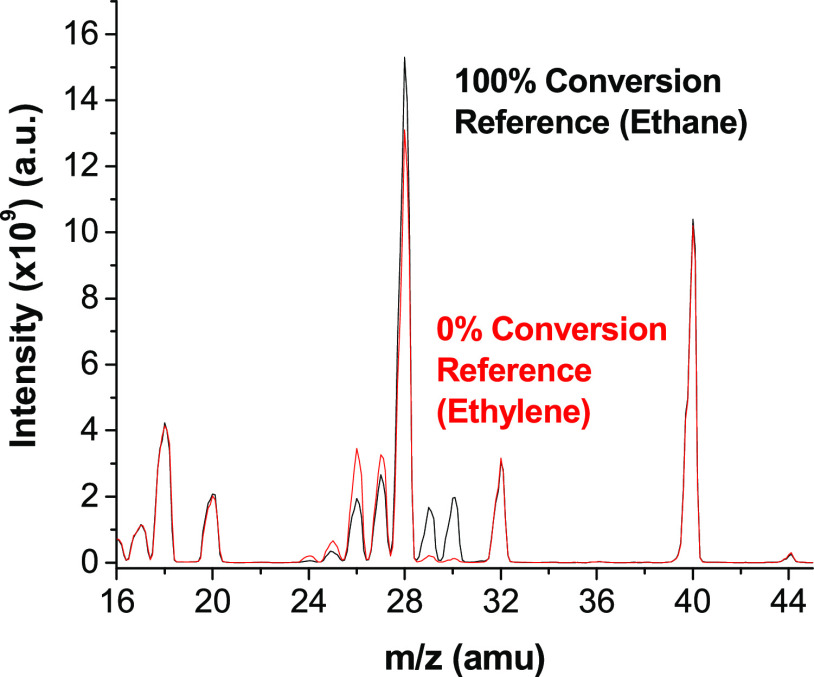
Mass spectra of the 0% conversion reference channel (red) and the 100% conversion reference channel (black) at room temperature with a reactant gas stream composed of *P*_H2_^in^ = 690 Torr, *P*_C2H4_^in^ = 25 Torr, *P*_O2_^in^ = 15 Torr, and *P*_Ar_^in^ = 30 Torr at a flow rate of 0.1 mL/min/channel. A comparison of the two spectra reveals that the differences between the signals can be attributed to the production of ethane (C_2_H_6_) in the 100% conversion reference channel, with increased signal intensities at *m*/*z* = 29 and 30 amu, accompanied by the consumption of ethylene and decreased signal intensities from *m*/*z* = 24 → 27 amu. No side reactions (e.g., combustion byproducts) are observed arising from the presence of O_2_ in the feed.

The extent of ethylene hydrogenation in the microreactor channels was determined by linear interpolation of the mass spectrometer signals at *m*/*z* = 29 and 30 amu (which correspond to the peaks of the product ethane molecule) between those of the 0 and 100% conversion reference channels. Figure 1 shows the mass spectra collected in the reference channels for a flow consisting of *P*_H2_^in^ = 690 Torr, *P*_C2H4_^in^ = 25 Torr, *P*_O2_^in^ = 15 Torr, and *P*_Ar_^in^ = 30 Torr at a total flow rate of 10 mL/min (∼0.1 mL/min/channel). The 0% conversion reference channel (red spectrum) gives the baseline signal of the reactant mixture from *m*/*z* = 16 → 45 amu, including small peaks at *m*/*z* = 29 and 30 amu corresponding to the natural abundance of ^13^C isotopes. The 100% conversion reference channel (black spectrum) delivers the reactant gases to the independently heated flow tube reactor loaded with enough Pd wire to reach 100% conversion of ethylene to ethane. Figure 1 shows that the only differences between the two reference channels are the appearance of signals at *m*/*z* = 29 and 30 amu associated with the production of ethane accompanied by a decrease in the intensity of the ethylene signals from *m*/*z* = 24 → 27 amu. Note that the signal at *m*/*z* = 28 amu is excluded from the analysis due to the back-diffusion of N_2_ from the air into the sampling tube. Inspection of the peaks at *m*/*z* = 18 amu (H_2_O), *m*/*z* = 32 amu (O_2_), and *m*/*z* = 44 amu (CO_2_) shows that they are the same intensity in both reference channels, indicating that nothing other than ethane (e.g., no combustion byproducts) was being produced during the reaction. For the data set collected without O_2_ in the feed, the reference channel spectra look identical to those in Figure 1, except for the absence of the O_2_ peak at *m*/*z* = 32 amu.

The signal intensities from the 0% conversion reference channel and the 100% conversion reference channel represent the extrema for the extent of the reaction under each set of experimental conditions. Therefore, the signal intensities measured inside the microreactor channels corresponding to ethylene and ethane (*m*/*z* = 24 → 30 amu) can, in principle, be linearly interpolated between those of the reference channels to find the fractional ethylene conversion or extent of reaction, ξ. The linearity of the mass spectrometer signal for *m*/*z* = 29 and 30 amu was confirmed by flowing different gas mixtures of C_2_H_4_, C_2_H_6_, and Ar corresponding to different extents of reaction, ξ, into the 0% conversion reference channel and measuring the signal intensity as a function of the emulated extent of reaction (see Figure S1). Since the greatest change in the mass spectra between ethylene and ethane occurs at *m*/*z* = 29 and 30 amu (Figure 1) and we have confirmed that these intensities are linear with respect to ξ (Figure S1), only those mass spectrometer signals were used when interpolating the reference channels to determine ξ inside each microreactor channel. It is important to note that as ξ increases from 0 to 1, there is a slight reduction in the total flow rate due to the consumption of two moles of reactants for every mole of ethane produced (i.e., H_2_ + C_2_H_4_ → C_2_H_6_). However, due to the low partial pressure of ethylene (*P*_C2H4_^in^ = 25 Torr) with respect to the total (*P*^tot^ = 760 Torr), the maximum reduction in the flow rate is only ∼3% when ξ = 1.

The data sets collected from these experiments consist of the ethylene conversion in each of the microreactor channels measured over all Ag_*x*_Pd_1–*x*_ compositions, reaction temperatures, and inlet hydrogen pressures, ξ(*x*,*T*,*P*_H2_^in^). In addition to characterizing the ethylene hydrogenation activity of the film, the main aim of this work is to demonstrate the influence of O_2_ at *P*_O2_^in^ = 15 Torr on the ethylene conversion across Ag_*x*_Pd_1–*x*_ composition space.

## 3 Results

### 3.1 Characterization of CSAF Composition

**Figure 2:**
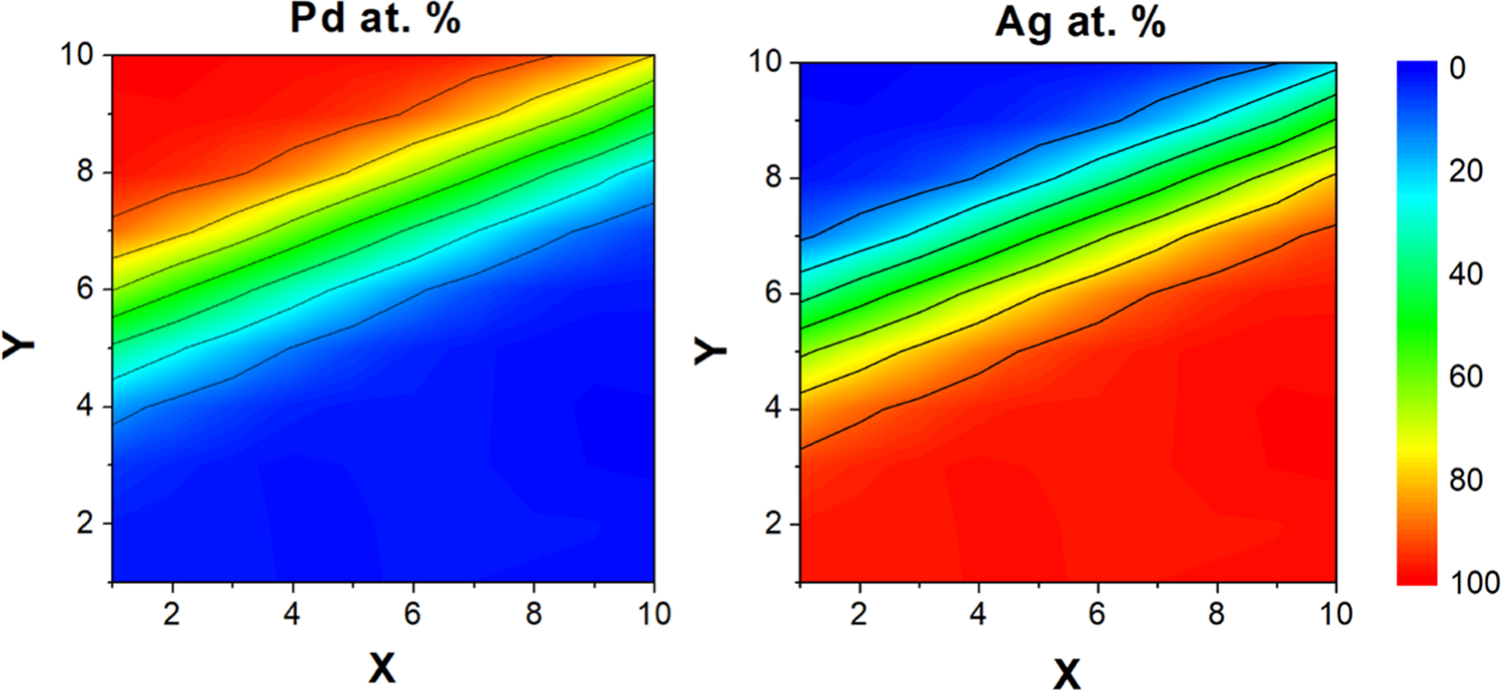
Bulk Ag_*x*_Pd_1–*x*_ composition maps as determined by EDX. The coordinates *X* and *Y* correspond to the column and row numbers, respectively, of the 10 × 10 array of independent flow microreactors.

The bulk composition and total thickness of the Ag_*x*_Pd_1–*x*_ CSAF were measured by EDX as a function of position using a 13 × 13 grid spanning the center of the Mo substrate with 1 mm spacing (Figure 2). The region of interest on the substrate is the 10 × 10 mm^2^ area spanned by the 10 × 10 array of independent flow reactors comprising the microreactor. Figure 2 shows the composition maps of Ag and Pd, where the ethylene hydrogenation activity was measured on the CSAF. The CSAF was deposited such that the isocomposition lines are oriented at an angle with respect to the edge of the substrate, which is aligned with the inlet and outlet channels of the glass microreactor block. In the region sampled by the microreactor, the Ag_*x*_Pd_1–*x*_ catalyst composition spanned the range *x*_Pd_ = 0 → 1 fairly uniformly.

### 3.2 Ethylene Hydrogenation Activity of Ag_*x*_Pd_1–*x*_ Alloys

**Figure 3:**
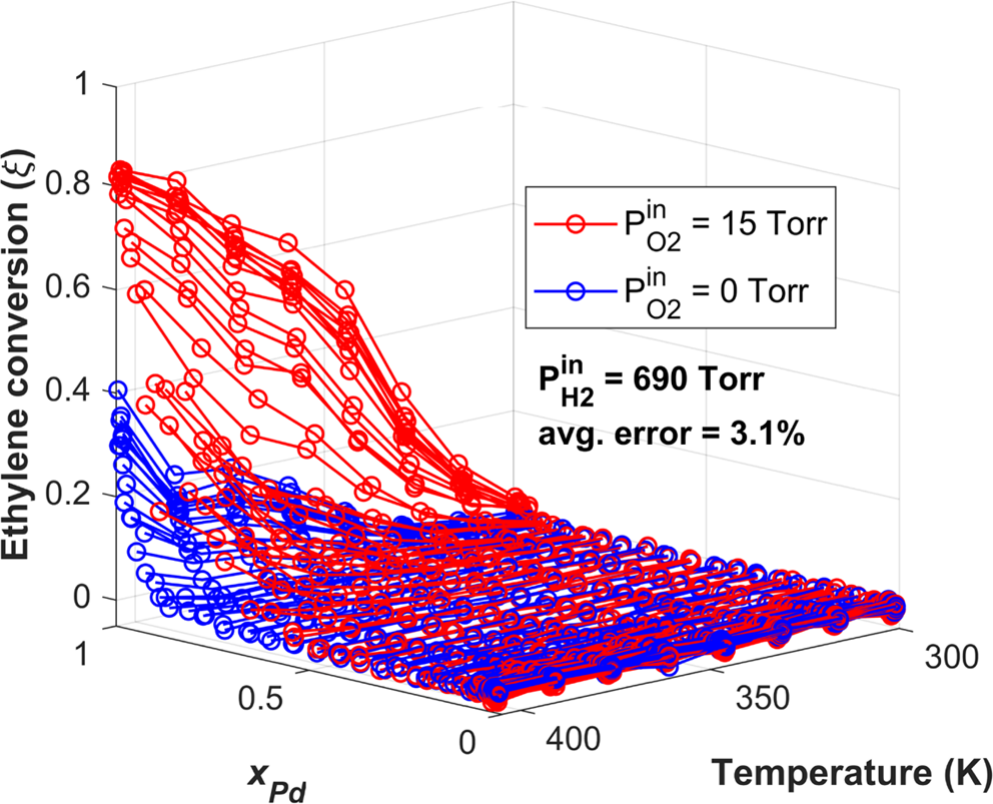
Ethylene conversion to ethane versus *x*_Pd_ and reaction temperature (K). The blue data set was obtained with *P*_O2_^in^ = 0 Torr, and the red data set was obtained with *P*_O2_^in^ = 15 Torr in the feed. The inlet partial pressures of the reactants were *P*_H2_^in^ = 690 Torr and *P*_C2H4_^in^ = 25 Torr, with the balance being Ar to achieve *P*^tot^ = 760 Torr and a total flow rate of 10 mL/min.

**Figure 4:**
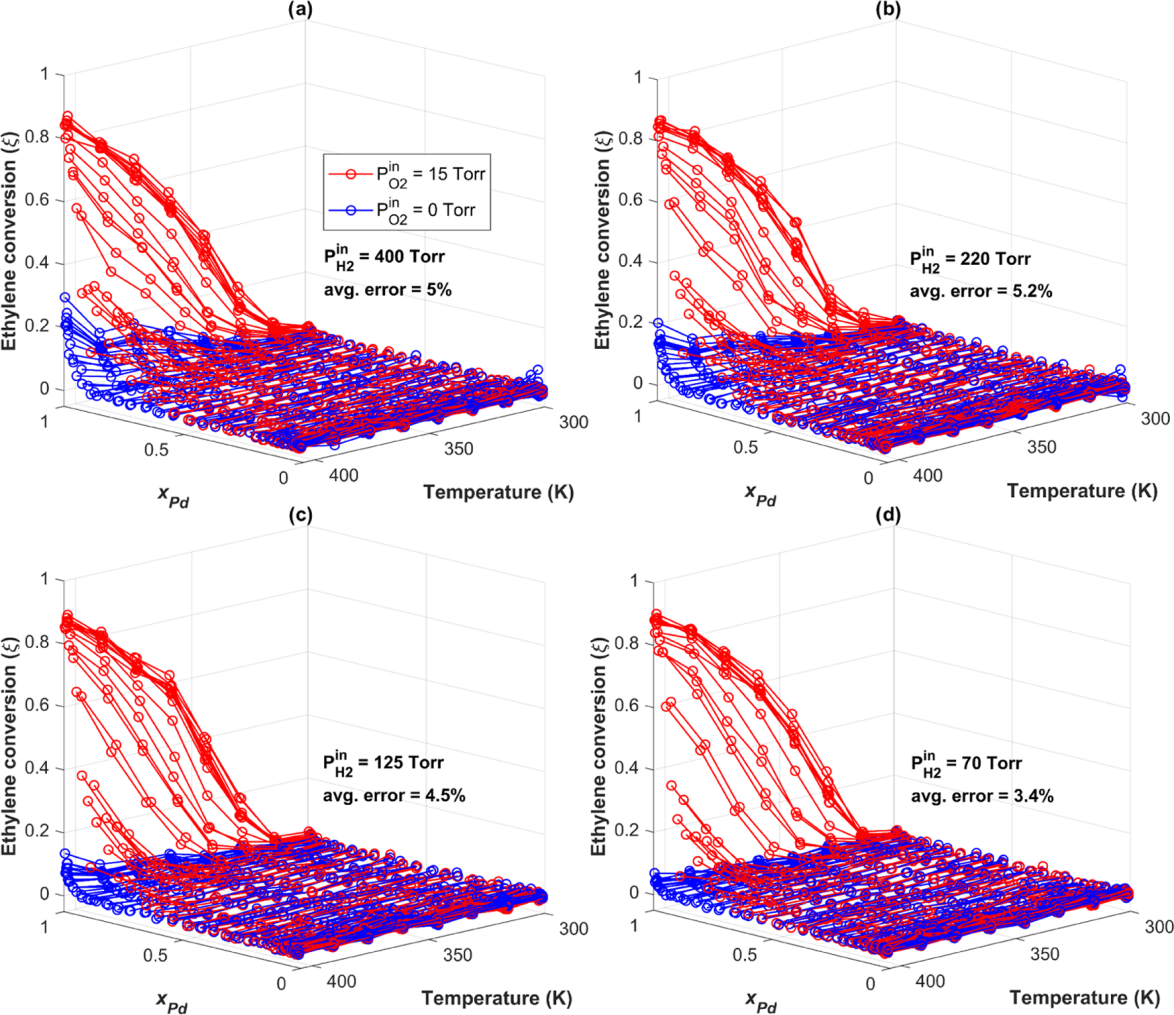
Ethylene conversion to ethane versus *x*_Pd_ and reaction temperature (K). The blue data sets were obtained with *P*_O2_^in^ = 0 Torr and the red data sets were obtained with *P*_O2_^in^ = 15 Torr in the feed. The different inlet hydrogen pressures, *P*_H2_^in^, are labeled in each graph along with the average percent error for all of the data with ξ > 0.03. In all cases, the inlet ethylene pressure was *P*_C2H4_^in^ = 25 Torr and the flow was balanced with Ar to achieve *P*^tot^ = 760 Torr and a total flow rate of 10 mL/min.

The ethylene hydrogenation activity of the Ag_*x*_Pd_1–*x*_ CSAF was measured by flowing H_2_, C_2_H_4_, O_2_, and Ar mixtures into the microreactor at a constant CSAF temperature, inlet pressure, and flow rate while measuring the product gas composition in the 100 outlets by mass spectrometry. Two sets of experiments were performed: one with *P*_O2_^in^ = 0 Torr and one with *P*_O2_^in^ = 15 Torr in the feed. Other than the presence/absence of O_2_, the flow conditions for the two data sets were identical since Ar was used to balance the total flow rate. In order to determine the extent of reaction, ξ, in each microreactor channel, the mass spectrometer signals at *m*/*z* = 29 and 30 amu were linearly interpolated between the signals from the reference channels having ξ = 0 and ξ = 1. The extents of ethylene hydrogenation were obtained for 100 different Ag_*x*_Pd_1–*x*_ alloy compositions spanning the range *x*_Pd_ = 0 → 1, at 8 different reaction temperatures from *T* = 300 → 405 K, and at 5 different hydrogen partial pressures from *P*_H2_^in^ = 70 → 690 Torr. The ethylene conversion at each set of experimental conditions (i.e., alloy composition, reaction temperature, and inlet hydrogen pressure) was measured in three sequential scans across Ag_*x*_Pd_1–*x*_ composition, spaced ∼20 min apart to confirm that the catalyst film displayed stable activity with no deactivation. In Figures 3 and 4, we report the average of the three ethylene conversion measurements with the average percent error for each data set calculated using only those conversions with ξ > 0.03.

Figure 3 shows the catalytic activity of the 100 Ag_*x*_Pd_1–*x*_ alloy compositions versus reaction temperature (K) for a feed composed of *P*_H2_^in^ = 690 Torr, *P*_C2H4_^in^ = 25 Torr, and *P*_Ar_^in^ = 45 Torr. As expected, the ethylene conversion increases as a function of both temperature and *x*_Pd_, with the maximum conversion occurring on the pure Pd catalyst at 405 K. As seen in Figure 3, when *P*_O2_^in^ = 0 Torr, no activity is observed for alloys with *x*_Pd_ < 0.9. Interestingly, when *P*_O2_^in^ = 15 Torr, alloys with as little as *x*_Pd_ = 0.6 become measurably active for the reaction and the ethylene conversion at Ag_*x*_Pd_1–*x*_ alloy compositions already activated at *P*_O2_^in^ = 0 Torr is significantly increased. For example, the maximum conversion achieved by the Pd-rich alloys at *T* = 405 K doubles from ξ ≈ 0.4 without O_2_ to ξ ≈ 0.8 when *P*_O2_^in^ = 15 Torr. Figure 4 shows the ethylene conversion versus *x*_Pd_ and reaction temperature with and without O_2_ in the feed for the four remaining inlet H_2_ partial pressures, spanning the range *P*_H2_^in^ = 400 Torr →70 Torr. Together, Figures 3 and 4 comprise the entire data set collected in these experiments, enabling the measurement of ethylene hydrogenation activity across all of Ag_*x*_Pd_1–*x*_ composition space from *T* = 300 K → 405 K and across an order of magnitude in *P*_H2_^in^. At all experimental conditions, the presence of 15 Torr of O_2_ in the feed causes a substantial increase in the ethylene conversion.

## 4 Discussion

The measured ethylene conversion on the Ag_*x*_Pd_1–*x*_ CSAF follows the expected activity trend with respect to alloy composition: high conversion for Pd-rich alloys and negligible conversion for Ag-rich alloys. The most active catalyst composition on the film was pure Pd, which is consistent with its high activity for H_2_ dissociation relative to that of Ag. The most interesting insight provided by these experiments comes from comparing the ethylene hydrogenation activity at the same alloy compositions with and without *P*_O2_^in^ = 15 Torr in the feed. The addition of O_2_ to the feed uniformly increases the activity across composition space at all reaction temperatures and inlet hydrogen pressures (Figures 3 and 4). For *P*_H2_^in^ = 690 Torr, the ethylene conversion on the pure Pd catalyst roughly doubles in the presence of O_2_. Figure 4 shows that this increase in activity becomes even more dramatic when *P*_H2_^in^ is decreased. The presence of O_2_ in the feed also lowers the bulk Pd concentration, *x*_Pd_, at which the onset of activity is observable. The use of the CSAF allowed us to map the effect of O_2_ on the composition-dependent catalytic behavior of the Ag_*x*_Pd_1–*x*_ binary system with unprecedented resolution in composition.

**Figure 5:**
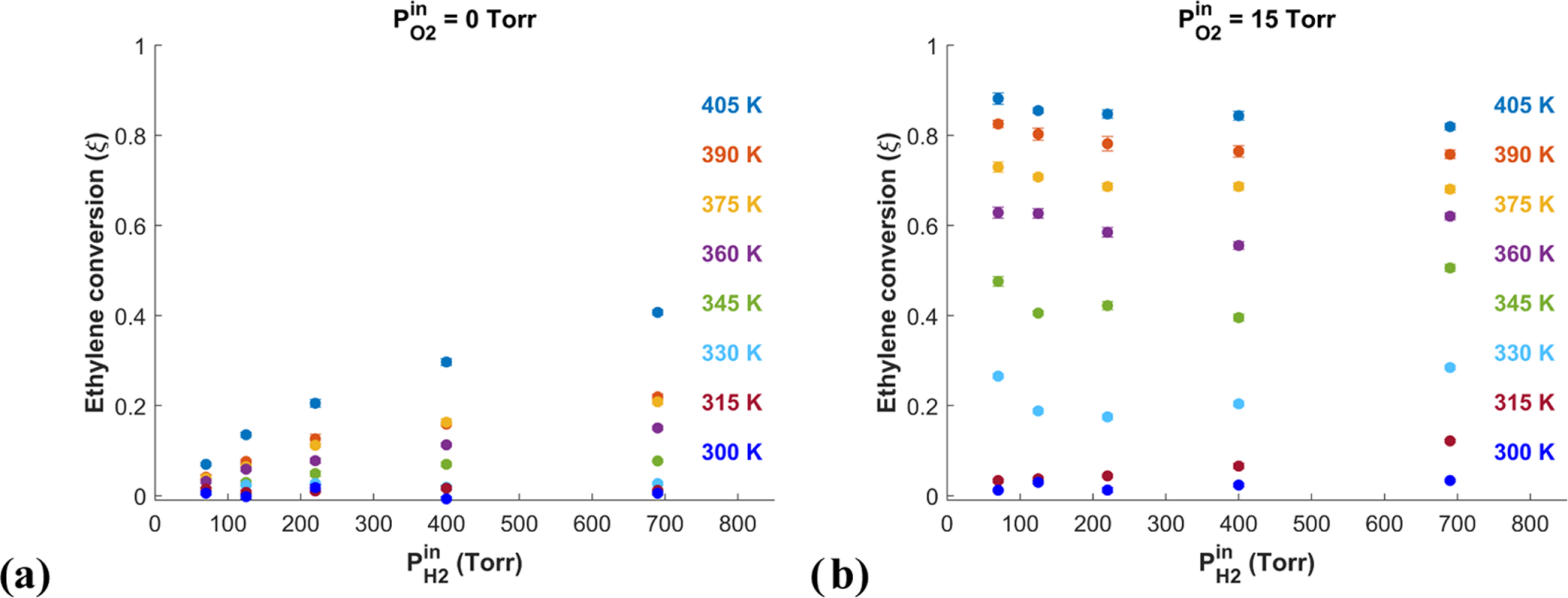
Ethylene conversion on the pure Pd catalyst versus *P*_H2_^in^ from *T* = 300 K → 405 K for a reactant feed composed of (a) *P*_C2H4_^in^ = 25 Torr and *P*_O2_^in^ = 0 Torr and (b) *P*_C2H4_^in^ = 25 Torr and *P*_O2_^in^ = 15 Torr. In both cases, Ar is used to balance the reactant mixture so that the total flow rate used in each experiment is constant. The presence of O_2_ in the reactant feed leads to an increase in the ethylene conversion at all *T* and *P*_H2_^in^.

Figure 5 highlights the influence of *P*_O2_^in^ on the ethylene hydrogenation activity of the pure Pd catalyst as a function of the inlet hydrogen pressure, *P*_H2_^in^, at all reaction temperatures. Figure 5a shows the ethylene conversion on pure Pd with *P*_O2_^in^ = 0 Torr, and Figure 5b shows the conversion with *P*_O2_^in^ = 15 Torr. The presence of O_2_ in the feed increases the ethylene conversion on Pd at all temperatures and inlet H_2_ pressures, *P*_H2_^in^, and this effect becomes more pronounced as *P*_H2_^in^ decreases. For example, at *P*_H2_^in^ = 70 Torr and *T* = 405 K, the presence of O_2_ raises the conversion from ξ < 0.1 to ξ ≈ 0.9. The extent of reaction is conversion-limited when *P*_O2_^in^ = 15 Torr but kinetically limited when *P*_O2_^in^ = 0 Torr. Using first-order kinetic analysis, the increase in conversion from 0.1 to 0.9 with the addition of O_2_ indicates an ∼20-fold increase in the intrinsic rate of ethylene hydrogenation. Since no Ag is present at this catalyst location, the dramatic increase in the catalytic activity can only be due to the interaction of O_2_ in the feed with Pd.

Comparing Figure 5a with 5b, one can observe a change in the conversion dependence on *P*_H2_^in^ when O_2_ is added to the feed. In the experiments performed with *P*_O2_^in^ = 0 Torr, the conversion at all temperatures appears to increase linearly with *P*_H2_^in^. By contrast, when *P*_O2_^in^ = 15 Torr, the conversion appears to be independent of *P*_H2_^in^ when *T* ≥ 330 K. This suggests that in the absence of O_2_, the surface sites are depleted in adsorbed H atoms, while in the presence of O_2_, they are saturated and therefore θ_H_ ≈ 1, so the surface H coverage (θ_H_) cannot be increased by increasing *P*_H2_^in^. In turn, this suggests that the addition of O_2_ results in a change to the reaction mechanism, or at least a change in the rate-limiting step of the mechanism resulting from the catalyst undergoing activation by O_2_.

To relate the ethylene conversion at each Ag_*x*_Pd_1–*x*_ catalyst composition with an energetic parameter describing the reaction, a pseudo-zero-order rate law was assumed for ethylene hydrogenation (eq 1) in the limit of low conversion. When the conversion of ethylene is low, which we define for this analysis as ξ ≤ 0.2, the change in the reactant concentration is negligible, and thus, the reaction rate can be approximated using only the rate constant, *k*. Equation 1 presents the zero-order rate law for ethylene hydrogenation, where the reaction rate, *r*, is given by a single rate constant, *k* (s^–1^), which can be found by dividing ξ at low conversion by the residence time of the gas mixture inside the microreactors, Δτ. In our system, the residence time Δτ = 0.17 s is well-defined since the volumetric flow rate through each reactor channel is set by the mass flow controllers, and the geometry of each reactor box is known (700 μm × 800 μm × 500 μm).
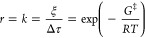
1Thus, from eq 1, a zero-order rate constant, *k*, can be found for a given extent of reaction, ξ, in the limit of low conversion and related to the free energy of activation for ethylene hydrogenation, *G*^‡^, where *R* is the ideal gas constant and *T* is the reaction temperature in K. Solving for *G*^‡^ in eq 1 yields eq 2, where all of the variables on the right-hand side are either constants or can be determined from the experimental data in Figures 3 and 4.
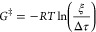
2

**Figure 6:**
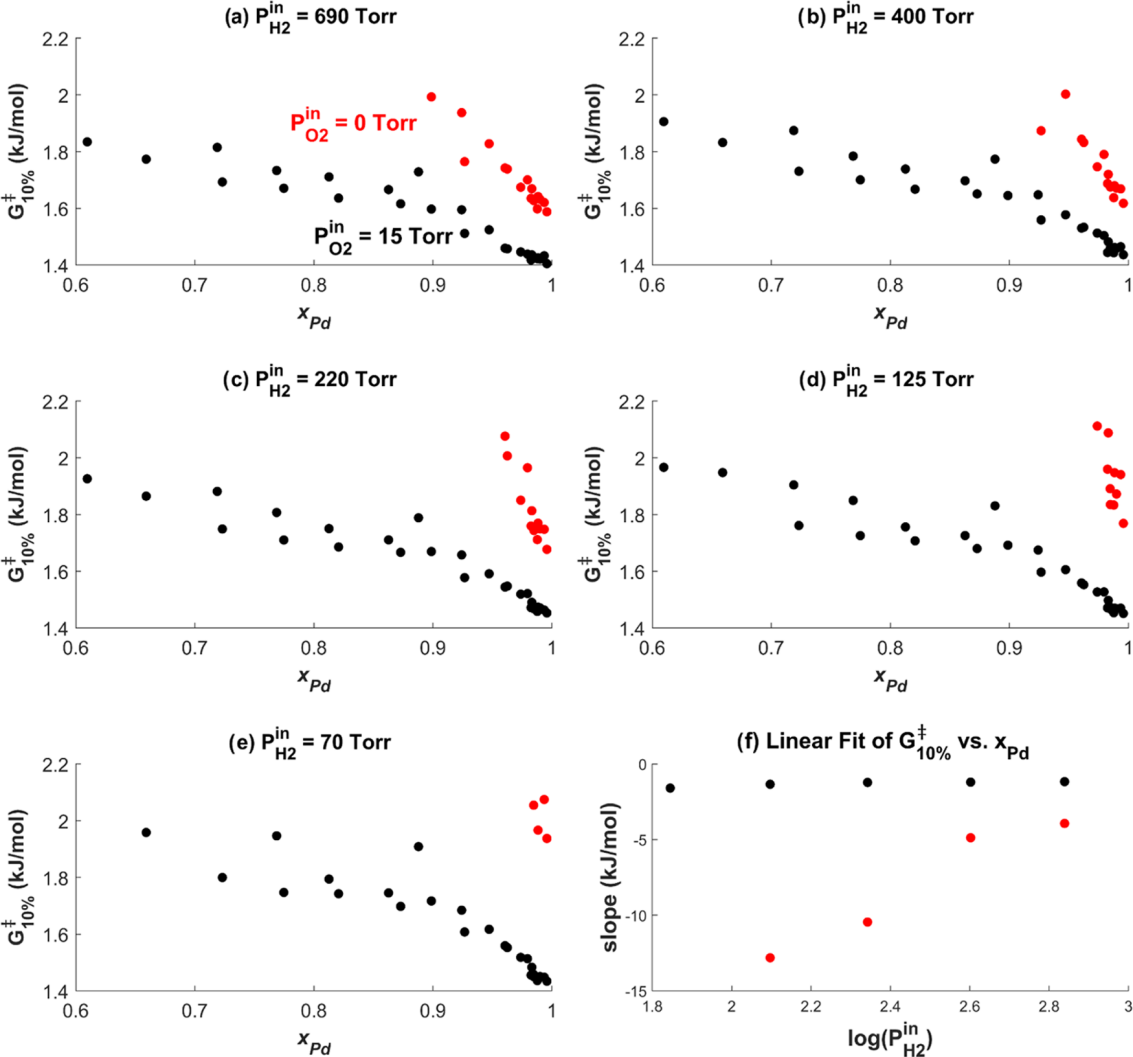
Estimated free energy of activation for 10% conversion of ethylene to ethane, *G*_10%_^‡^, versus *x*_Pd_ with *P*_O2_^in^ = 0 Torr (red points) and *P*_O2_^in^ = 15 Torr (black points) in the feed for *P*_H2_^in^ = 690 Torr →70 Torr (a) through (e), respectively. *G*_10%_^‡^ was calculated by fitting a quadratic to the low conversion data (ξ ≤ 0.2) in Figures 3 and 4 to determine the relationship for ξ(*T*) for each alloy composition and combination of inlet partial pressures and then using the value of *T* at ξ = 0.1 in eq 2. When *P*_O2_^in^ = 0 Torr (red points), estimates of *G*_10%_^‡^ are only available for catalysts with *x*_Pd_ > 0.9, and as *P*_H2_^in^ decreases, fewer compositions are sufficiently active to be fit using a quadratic (i.e., less than 3 data points with ξ > 0). When *P*_O2_^in^ = 15 Torr (black points), *G*_10%_^‡^ is reduced for all *P*_H2_^in^ and can be calculated for alloys with as little as *x*_Pd_ > 0.6 due to the activity boost across the CSAF caused by the presence of O_2_. The slopes of the lines of best fit for (a–e) are plotted in (f) versus log(*P*_H2_^in^), highlighting that *P*_H2_^in^ causes a negligible change in the dependence of *G*_10%_^‡^ on *x*_Pd_ when *P*_O2_^in^ = 15 Torr but that the dependence is highly sensitive to *P*_H2_^in^ when *P*_O2_^in^ = 0 Torr. This suggests a change in the reaction mechanism (or at least the rate-limiting step) depending on whether or not O_2_ is present. Note that the slope from fitting the data in (e) when *P*_O2_^*in*^ = 0 Torr was omitted from (f) due to the large uncertainty resulting from the relatively small number of points.

The low conversion data in Figures 3 and 4 were fit using a quadratic to determine the relationship between ξ and *T* at each Ag_*x*_Pd_1–*x*_ composition. Using the equation describing ξ(*T*) at each alloy composition, we then estimated the value of *T*_10%_, the temperature needed to reach 10% ethylene conversion (ξ = 0.1), and used these values in eq 2 to calculate *G*^‡^ for each set of experimental conditions (i.e., alloy composition, *P*_O2_^in^, and *P*_H2_^in^). Figure 6a–e shows *G*_10%_^‡^, the free energy of activation to achieve ξ = 0.1, versus *x*_Pd_ for all inlet H_2_ pressures, *P*_H2_^in^, used in the reactant feed. This figure allows us to investigate how the gradual dilution of Pd with Ag affects the energetics of the catalyst by comparing all of the compositions and reaction conditions at the same level of activity.

Figure 6a–e shows *G*_10%_^‡^ versus *x*_Pd_ for all of the inlet H_2_ pressures, *P*_H2_^in^, used in the reactant feed. When *P*_O2_^in^ = 0 Torr (the red data points), *G*_10%_^‡^ can only be calculated for alloys with *x*_Pd_ ≥ 0.9 due to low activity, while *G*_10%_^‡^ can be calculated for alloys with as little as *x*_Pd_ = 0.6 when *P*_O2_^in^ = 15 Torr (the black data points). At all values of *P*_H2_^in^ and *P*_O2_^in^, *G*_10%_^‡^ decreases to its minimum as *x*_Pd_ → 1, which supports the understanding that the barrier to ethylene hydrogenation is lower on pure Pd than on AgPd alloys, as evidenced by its higher activity. When *P*_O2_^in^ = 0 Torr and *P*_H2_^in^ = 690 Torr (Figure 6a), pure Pd (i.e., *x*_Pd_ = 1) has *G*_10%_^‡^ ≈ 1.6 kJ/mol, and as *P*_H2_^in^ is decreased to *P*_H2_^in^ = 70 Torr (Figure 6e), we see an increase in *G*_10%_^‡^ to ∼2 kJ/mol. The negative dependence of *G*_10%_^‡^ on *P*_H2_^in^ indicates that ethylene hydrogenation is less favorable when fewer H_2_ molecules are present in the reactant feed, presumably due to the competition for adsorption sites. On the other hand, when *P*_O2_^in^ = 15 Torr, *G*_10%_^‡^ decreases to ∼1.4 kJ/mol for pure Pd and is relatively insensitive to changes in *P*_H2_^in^, which we attribute to the increased activity of PdO_*x*_ formed in the presence of O_2_ (explained in more detail below).

From Figure 6, we can also investigate the negative dependence of *G*_10%_^‡^ on *x*_Pd_ to highlight differences arising from *P*_O2_^in^. At both *P*_O2_^in^ = 0 Torr and *P*_O2_^in^ = 15 Torr, *G*_10%_^‡^ decreases as *x*_Pd_ increases until it reaches its minimum at *x*_Pd_ = 1. The increase in *G*_10%_^‡^ as *x*_Pd_ decreases can be understood when considering that higher free energy of activation is necessary for the reaction to occur due to the degradation in catalytic activity as more Ag is present in the bulk. When *P*_O2_^in^ = 15 Torr, *G*_10%_^‡^ increases gradually by ∼30% from 1.4 to ∼2 kJ/mol as *x*_Pd_ decreases from 1 → 0.6, below which the activity of the film is not measurable.

To observe the effect of *P*_H2_^in^ on the relationship between *G*_10%_^‡^ and *x*_Pd_, the slope of the lines of best fit for Figure 6a–e are plotted versus log(*P*_H2_^in^) in Figure 6f. Note that the slope of the line of best fit at *P*_H2_^in^ = 70 Torr and *P*_O2_^in^ = 0 Torr (the red data set in Figure 6e) was omitted from Figure 6f due to the large uncertainty resulting from fitting with a relatively small number of points. From Figure 6f, we can conclude that when *P*_O2_^in^ = 15 Torr, *P*_H2_^in^ causes no meaningful change in the relationship between *G*_10%_^‡^ and *x*_Pd_. In other words, at all *P*_H2_^in^, *G*_10%_^‡^ increases at the same rate from its minimum value of ∼1.4 kJ/mol at *x*_Pd_ = 1 to ∼2 kJ/mol at *x*_Pd_ ≈ 0.6. On the other hand, when *P*_O2_^in^ = 0 Torr, the slope of *G*_10%_^‡^ versus *x*_Pd_ is highly sensitive to *P*_H2_^in^, becoming much more negative as *P*_H2_^in^ decreases. This means that when *P*_O2_^in^ = 0 Torr, the energetics of ethylene hydrogenation become highly unfavorable when pure Pd is diluted with even trace amounts of Ag and this effect becomes more pronounced when less H_2_ is present in the reactant stream. Thus, not only is *G*_10%_^‡^ systematically higher at all alloy compositions when *P*_O2_^in^ = 0 Torr, but it is much more sensitive to small changes in *x*_Pd_ than when *P*_O2_^in^ = 15 Torr. Analysis of Figure 6 provides support for the activity trends observed in Figures 3–5 using an energetic parameter that describes the activation of the catalyst for ethylene hydrogenation, *G*_10%_^‡^. The systematic decrease in *G*_10%_^‡^ and its decreased sensitivity to both *x*_Pd_ and *P*_H2_^in^ when O_2_ is present in the feed highlight the sensitivity of AgPd catalyst performance to the feed conditions.

**Figure 7:**
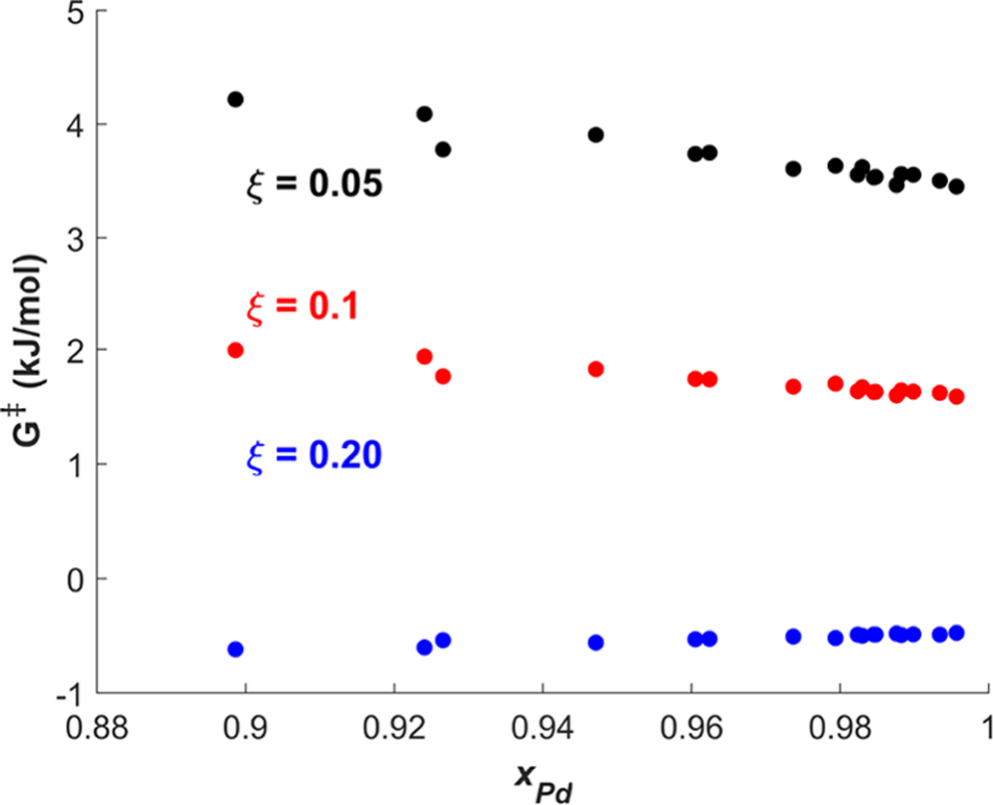
Calculated free energy of activation, *G*^‡^, when ethylene hydrogenation achieves ξ = 0.05 (black data points), ξ = 0.1 (red data points), and ξ = 0.2 (blue data points) versus *x*_Pd_ for a reactant feed composed of *P*_H2_^in^ = 690 Torr and *P*_O2_^in^ = 0 Torr. *G*^‡^ was calculated using the relationship for ξ(*T*) found by fitting the activity data in Figure 3 to a quadratic equation and inputting the estimated reaction temperature, *T*, to reach the desired ξ into eq 2. Note that the red data points for ξ = 0.1 are identical to the red data points in Figure 6a. At each ξ, *G*^‡^ displays a slight dependence on *x*_*Pd*_ within the range 0.9–1, as in Figure 6. As ξ increases, *G*^‡^ decreases from ∼4 kJ/mol at ξ = 0.05 to ∼2 kJ/mol at ξ = 0.1, to ∼ −0.5 kJ/mol at ξ = 0.2. Such a minor offset in *G*^‡^ (±2 kJ/mol) falls within the expected uncertainty for the energetics of the reaction.

To check our assumption of a pseudo-zero-order rate law for ethylene hydrogenation, *G*^‡^ was calculated for other extents of reaction for a feed composed of *P*_H2_^in^ = 690 Torr and *P*_O2_^in^ = 0 Torr and then plotted in Figure 7 versus *x*_Pd_. *G*^‡^ at each alloy composition was found by using the relationship for ξ(*T*) derived from the quadratic fit used to obtain Figure 6 and then interpolating to other extents of reaction. Note that the red data points in Figure 7 for ξ = 0.1 are identical to the red data points in Figure 6a. Note also that the dependence of *G*^‡^ on *x*_Pd_ is difficult to see when all three values of ξ are plotted on the same figure; however, modification of the *y*-axis shows that there is a similar dependence on *x*_Pd_ as in Figure 6. As ξ increases, *G*^‡^ decreases uniformly from ∼4 kJ/mol at ξ = 0.05 to ∼2 kJ/mol at ξ = 0.1 and finally to ∼ −0.5 at ξ = 0.2. Such a minor offset in *G*^‡^ (±2 kJ/mol) at different extents of reaction is within the expected uncertainty for the energetics of the catalyst. Therefore, in the limit of low conversion, *G*^‡^ is a useful energetic parameter for describing the activation of Ag_*x*_Pd_1–*x*_ catalysts for ethylene hydrogenation.

In justifying the dramatic boost in activity caused by the presence of O_2_ in the feed, the first question to address is whether O_2_ just activates the Ag_*x*_Pd_1–*x*_ catalysts or participates in undesirable side reactions. There is no evidence of O_2_ consumption via combustion with either H_2_ or ethylene. The mass spectra in Figure 1 show no difference between the signal of O_2_ at *m*/*z* = 32 amu measured in the 100% conversion reference channel and the 0% conversion reference channel (feed gas signal). Furthermore, there are no increases in the signals at either *m*/*z* = 18 amu (H_2_O) or *m*/*z* = 44 amu (CO_2_) in the 100% conversion reference channel, i.e., there is no sign of catalytic combustion.

The most likely explanation for the increased ethylene hydrogenation activity of Ag_*x*_Pd_1–*x*_ alloys in the presence of O_2_ is a combination of enhanced Pd segregation in the presence of O_2_ and simply the fact that oxidized or otherwise O-modified Pd is a more effective catalyst for ethylene hydrogenation than metallic Pd. Palladium oxide, PdO, has already been shown to be effective for catalyzing the combustion of organic compounds,^55,56^ the dimerization of methane,^57^ and the methanation of CO_2_.^58^ However, to our knowledge, PdO has never explicitly been used for catalytic hydrogenation, despite the widespread use of oxide-supported Pd.^59−61^ Our results show a clear enhancement of the ethylene hydrogenation activity in the presence of O_2_, even for the pure Pd catalyst. Thus, the results of this work show the potential of using O_2_ to modify and activate Pd for catalytic processes involving hydrogenation reactions. Note that characterization of the catalyst surface during and after the reaction was not possible due to the contamination that would have occurred when transferring the CSAF through the air. As a result, we can only make inferences about the nature of the activated Pd catalysts using the activity data when O_2_ is included in the feed and expectations for the behavior of AgPd binary alloys in O_2_ established in the literature. Consequently, the extent of the interaction between O_2_ and Pd, whether through bulk oxidation and/or surface modification through O chemisorption potentially leading to the formation of OH groups, remains unspecified.

**Figure 8:**
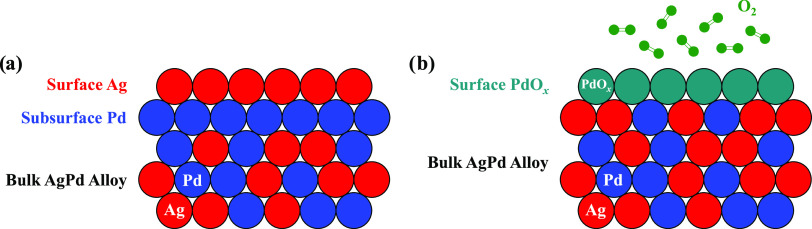
Graphical illustration of the Ag_*x*_Pd_1–*x*_ alloy surface with (a) *P*_O2_^in^ = 0 Torr and (b) *P*_O2_^in^ = 15 Torr in the reactant feed. When *P*_O2_^in^ = 0 Torr, Ag atoms cover the top surface to minimize the total surface free energy. Consequently, Pd atoms in the subsurface and bulk are inaccessible to adsorbates and cannot catalyze ethylene hydrogenation. On the other hand, when *P*_O2_^in^ = 15 Torr, interactions between Pd and O_2_ draw the Pd atoms to the top surface, both increasing and activating new sites for the reaction. The restructuring of the catalyst surface facilitated by the presence of O_2_ is the proposed explanation for why the catalytic activity across the CSAF changes so dramatically when *P*_O2_^in^ = 15 Torr.

In addition to the possibility that O_2_ activates Pd to form a more active catalyst, herein denoted as PdO_*x*_, another explanation for the enhancement in the ethylene conversion of binary Ag_*x*_Pd_1–*x*_ alloys is an O_2_-mediated segregation of Pd to the top surface. Both computational and experimental studies have shown that in vacuum, Ag tends to segregate to the top surface in AgPd binary systems (Figure 8a) because of its lower surface free energy relative to that of Pd.^47,62−64^ On otherwise equivalent FCC(111) slabs,^40^ calculations have shown that Ag has a surface free energy of γ_Ag_ = 1.2 J/m^2^, while that of Pd is γ_Pd_ = 1.6 J/m^2^. One experimental study has even found that the barrier for Ag migration to cover Pd on the top surface is so low that it occurs in just minutes at room temperature when Pd is present in discontinuous islands across the surface.^65^ The Ag enrichment created by the surface free energy differential between Ag and Pd presumably passivates the catalytic activity for ethylene hydrogenation at the surface. However, in the presence of adsorbates, such as O_2_, it has been observed that preferential binding of the chemisorbed species to Pd will induce a driving force for Pd to segregate to the top surface, replacing Ag.^43,47,63,64^ Since O_2_ enhancement of activity is also observed for pure Pd, which is not subject to segregation, it seems likely that both Pd segregation and Pd activation by O_2_ are at play in the observed activity change for the Ag_*x*_Pd_1–*x*_ catalysts.

One relevant study conducted by van Spronsen et al. combined experimental and computational methods to investigate the restructuring of Pd deposited on a Ag(111) single crystal induced by O_2_ and CO adsorbates.^47^ In that study, after annealing the Pd-coated Ag(111) for several minutes in UHV at 400 K, they found that the most stable configuration of the alloy was a Ag-capped surface, even when Ag(111) was initially covered by a monolayer of Pd. Exposure of the Ag-capped surface to 1 Torr of O_2_ at 400 K was sufficient to resegregate Pd to the surface, as measured using ambient pressure XPS. The Ag_*x*_Pd_1–*x*_ CSAF used in this work is comparable to the AgPd alloy system studied by van Spronsen et al. The conditions used for those experiments on the Pd-coated Ag(111) were between 300 and 425 K for periods of minutes, quite similar to the temperatures used for ethylene hydrogenation in this work. Figure 8a shows a plausible rendering of the surface of our Ag_*x*_Pd_1–*x*_ alloys under the conditions where *P*_O2_^in^ = 0 Torr. When the bulk composition of Ag is sufficiently high, Ag atoms saturate the top surface to minimize the surface free energy and leave no active Pd atoms available to participate in ethylene hydrogenation. This likely explains why only those Ag_*x*_Pd_1–*x*_ alloys with *x*_Pd_ ≥ 0.9 displayed activity in the experiments performed with *P*_O2_^in^ = 0 Torr. Presumably, a bulk composition of *x*_Pd_ ≥ 0.9 ensures that some catalytically active Pd atoms are present on the top surface despite the inherent driving force to form the Ag-capped surface seen in Figure 8a.

**Figure 9:**
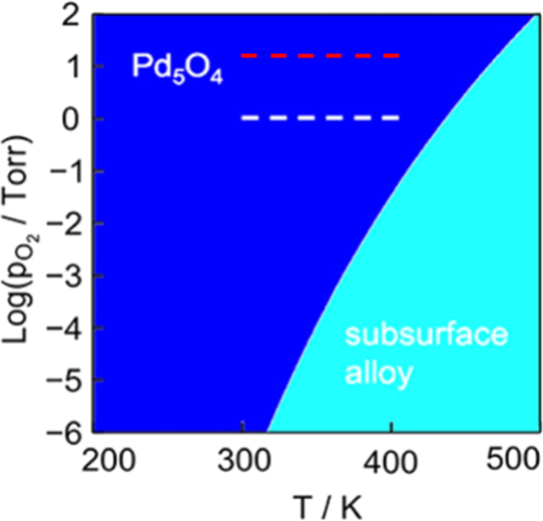
Regions of stability for the Pd_5_O_4_/Ag(111) surface oxide and the Ag-capped subsurface alloy as a function of temperature and O_2_ pressure calculated using DFT. The Pd_5_O_4_/Ag(111) surface oxide is predicted to be stable at high O_2_ pressures and/or low temperatures. The experimental conditions used by van Spronsen et al. for ambient pressure XPS measurements^47^ are marked by the white dashed line, and the experimental conditions used in this work (*P*_O2_^in^ = 15 Torr and *T* = 300 K → 405 K) are marked by the red dashed line. Figure reproduced with permission from ref (47). Copyright 2019 American Chemical Society.

On the other hand, we propose that when *P*_O2_^in^ = 15 Torr, the Ag_*x*_Pd_1–*x*_ alloys were restructured due to interactions between O_2_ and Pd, causing the latter to re-emerge on the surface and leading to substantial increases in ethylene hydrogenation activity, presumably because of the formation of activated PdO_*x*_ species. The resurfacing and activation of Pd atoms in the presence of O_2_ to create a new top surface is represented schematically in Figure 8b. Comparison of Figure 8a with 8b shows how a Ag_*x*_Pd_1–*x*_ alloy with the same bulk composition can have functionally different surfaces based on the operating conditions used. The idea that a relatively modest O_2_ pressure can cause such a strong effect on the surface structure of AgPd catalysts is supported by the computational predictions of van Spronsen et al.^47^ Using DFT, they calculated the regions of stability for the formation of a Pd surface oxide (which they proposed was Pd_5_O_4_) and a Ag-capped “subsurface alloy” (i.e., where no Pd resides on the surface) based upon the ambient temperature and the O_2_ pressure (Figure 9). It is important to note that the Ag-capped subsurface alloy and the Pd_5_O_4_ oxide shown in Figure 9 are analogous to the structures presented in Figure 8a and 8b, respectively. DFT calculations predicted the formation of the Pd surface oxide at high O_2_ pressures and/or low temperatures. In Figure 9, the experimental conditions used for ethylene hydrogenation in our experiments, with *P*_O2_^in^ = 15 Torr and *T* = 300 → 405 K, are marked by the red dashed line. Presumably, the line representing the experiments performed with *P*_O2_^in^ = 0 Torr would be vertically shifted below the axis in Figure 9 into the region where only the Ag-capped subsurface alloy is energetically favorable, consistent with our catalytic activity measurements. The robust ethylene hydrogenation activity across the CSAF when *P*_O2_^in^ = 15 Torr is contained exclusively in the region where the Pd surface oxide is predicted to form.

One important difference between our experiments and the results obtained by van Spronsen et al. in Figure 9 is the presence of a fixed H_2_ pressure in the feed between 70 and 690 Torr. Since H_2_ is always present during our experiments at a pressure greater than O_2_ (fixed at 15 Torr), it is not clear that Pd will be oxidized in such an environment. In essence, we have a competition between the oxidation and reduction of Pd, and it is not clear a priori what the stable phase should be under these conditions. While we have found no studies that conclusively characterize the stable phase of Pd under conditions where both H_2_ and O_2_ are present in such quantities, a relevant work by Kitchin et al. calculates the configurations of Ag_3_Pd(111) in oxygen atmospheres using first-principles thermodynamic calculations to establish trends in segregation, adsorption, and surface free energies of the alloy system.^66^ The first important result is that their calculations are consistent with the graphic illustration presented in Figure 8a, namely, that the most stable surface configuration of AgPd systems in the absence of O_2_ is a Ag-terminated alloy, stabilized by its lower surface free energy, with 100% Ag in the first layer and 100% Pd in the second layer.^66^ Their calculations also predict that the stronger binding of O_2_ to Pd causes O_2_-induced Pd segregation to replace Ag on the top surface of the alloy. More precisely, segregating Pd atoms to the top layer allows direct coordination of Pd with O adsorbates, causing the average binding energy to come into the same range as the average binding energy for O atoms on Pd(111), −1.29 eV/O atom,^66^ which implies a configuration with 100% Pd in the first layer and 100% Ag in the second layer. However, Kitchin et al. note that stabilizing such configurations involves the high energy cost of segregating Pd to the topmost layer and thus delays their stability range up to O_2_ chemical potentials where bulk oxide formation is already about to set in.^66^ In other words, O–Pd binding is not stronger than O–Ag binding by a sufficient amount to cause adsorbate-induced segregation unless the driving force from the gas phase is already so high that it directly initiates the formation of bulk-like PdO films at the surface.^66^ Thus, one possible scenario is that the boost in catalytic activity observed in Figures 3 and 4 due to the presence of O_2_ results from the simultaneous segregation and oxidation of Pd atoms. However, this does not preclude the possibility that other surface species are still possible, such as the formation of OH groups atop an oxidized Pd surface, especially given the high *P*_H2_^in^ used in our experiments.

In addition to thermodynamic support for the formation of a bulk-like PdO film at the topmost surface of AgPd alloys in the presence of a sufficiently high O_2_ partial pressure, there is also evidence that hydrogen migration in oxidized AgPd systems dramatically enhances the reduction of Ag over Pd. A relevant experimental study used ambient pressure XPS measurements to compare the reduction rate of oxidized Ag(111) in H_2_ with oxidized Ag(111) onto which Pd had been deposited.^67^ It is worthwhile to note that the oxidation conditions used in that study were 3 Torr of O_2_ at 373 K for 25 min, i.e., not as strong as the conditions used in our experiments (a steady flow containing 15 Torr of O_2_ between 300 and 405 K for >2 h). In the XPS study, it was found that the presence of PdO enhanced the rate of reduction of Ag surface oxide by more than 10^4^, which was attributed to rapid H_2_ dissociation on PdO followed by migration of atomic H across the Ag–Pd interface and reduction of Ag oxide.^67^ On the other hand, the net rate of reduction of PdO is thought to be mitigated by the migration of atomic O from rapidly reduced Ag oxide to partially reduced PdO.^67^ Thus, the implications for the current work suggest that in our experiments where both H_2_ and O_2_ are present in the feed, Pd is most likely to be oxidized and Ag is most likely to be reduced, although this does not preclude other surface modifications caused by chemisorbed O. The same study also observed an increase in the catalytic surface area of Pd upon oxidation, as the deposited Pd “islands” increased from monolayers with an average height of 0.2 nm to multilayers with an average height of 0.9 nm upon exposure to 3 Torr of O_2_ at 373 K for 25 min.^67^ While the exposed surface area of our AgPd catalysts were fixed by the dimensions of the gasket (700 × 800 μm^2^), some surface roughening accompanying the activation and surface segregation of Pd might have served to increase the reactivity even further.

Consequently, we conclude that the substantial boost in the catalytic activity of the Ag_*x*_Pd_1–*x*_ CSAF arises from the simultaneous surface segregation and activation of Pd atoms due to their favorable interactions with the O_2_ in the feed. Our results indicate that the driving force for Pd surface segregation and activation in the range *T* = 300 K → 405 K becomes diminished in AgPd alloys with a bulk composition of *x*_Pd_ < 0.6. In this case, high Ag composition prevents the preferential segregation of Pd atoms due to the high energy cost of surface rearrangement, leaving them buried beneath an entirely Ag-capped surface and, therefore, inaccessible for catalysis.

To support the proposed mechanism by which O_2_ affects the behavior of the Ag_*x*_Pd_1–*x*_ catalysts, we have estimated the reaction order with respect to hydrogen, *n*_H2_, across the CSAF with *P*_O2_^in^ = 0 Torr and with *P*_O2_^in^ = 15 Torr. As defined in eq 3, *n*_H2_ can be estimated using the measured ethylene conversion, ξ, since it is proportional to the total rate of ethane production, *r*_C2H6_, in the limit of low conversion. Thus, finding the change in log(ξ) with respect to log(*P*_H2_^in^) enables us to approximate *n*_H2_ for each alloy catalyst at each set of experimental conditions.
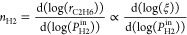
3

Estimates of *n*_H2_ were found by plotting log(ξ) versus log(*P*_H2_^in^) for each set of experimental conditions and finding the slope of the line of best fit. Figure S2 presents a subset of the plots of log(ξ) versus log(*P*_H2_^in^) to show which data points were included in the calculation of *n*_H2_ for the 20 most Pd-rich alloys ranging from *x*_Pd_ = 1 → 0.86 under conditions where *P*_O2_^in^ = 15 Torr and *T* = 375 K → 330 K. In brief, only those data points with low conversion above the noise level, defined as 0.02 < ξ < 0.3, were used for the analysis. A linear fit was determined to be the most appropriate on the basis of the appearance of the data in Figure S2 and the expectation that *n*_H2_ should not change with *P*_H2_^in^ on Pd surfaces. Thus, from the slope of the best-fit line of log(ξ) versus log(*P*_H2_^in^), we obtain the average value of *n*_H2_ over an order of magnitude change in *P*_H2_^in^. Next, we investigate how *n*_H2_ changes with Ag_*x*_Pd_1–*x*_ catalyst composition and *P*_O2_^in^ to help explain the activity differences measured across the CSAF.

**Figure 10:**
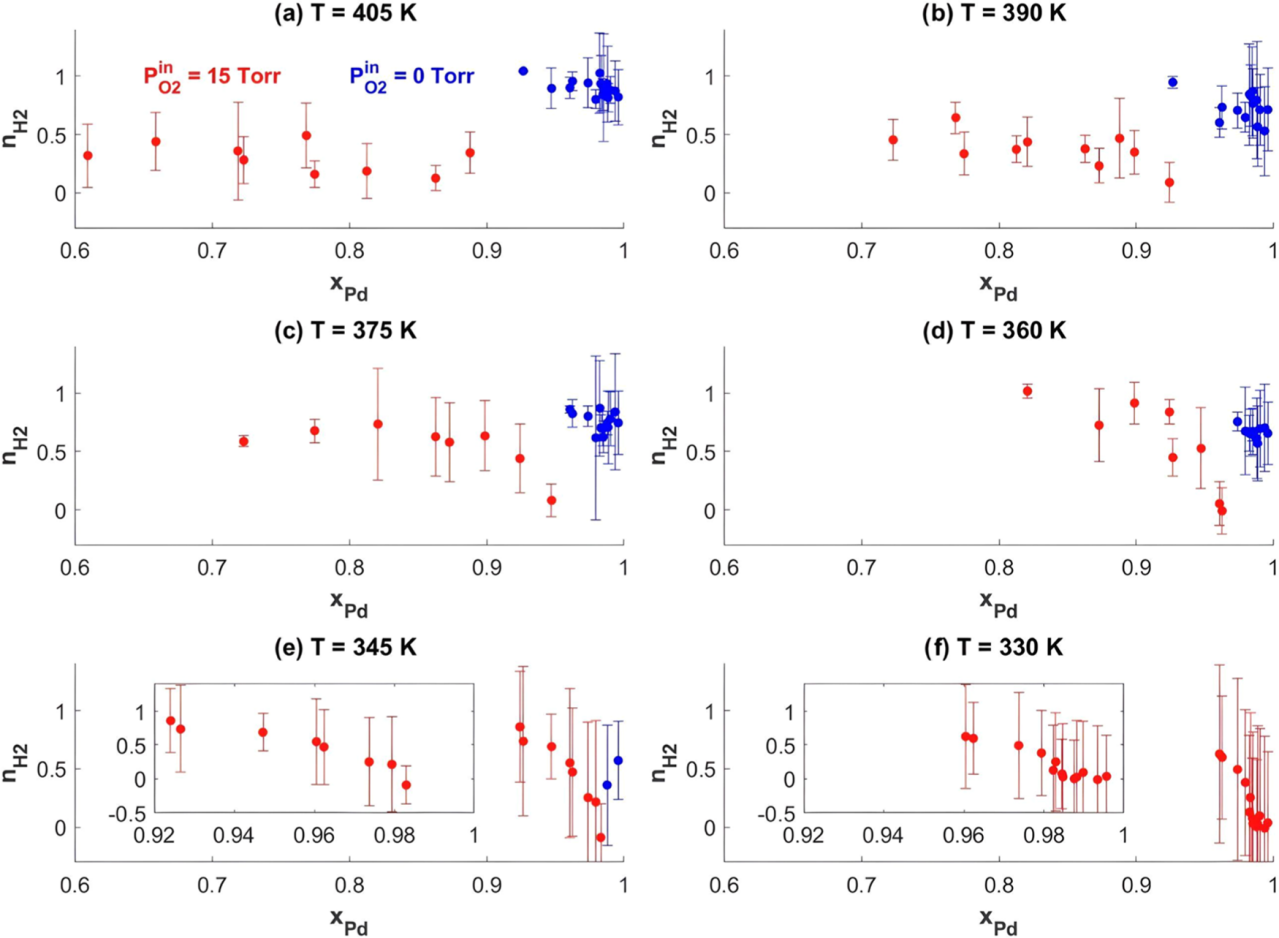
Calculated *n*_H2_ versus *x*_Pd_ for *T* = 405 K → 330 K, (a) through (f), respectively, for *P*_O2_^in^ = 0 Torr (blue) and *P*_O2_^in^ = 15 Torr (red). Due to the low activity across the CSAF when *P*_O2_^in^ = 0 Torr, *n*_H2_ can only be estimated for alloys with *x*_Pd_ > 0.92 at *T* = 405 K, and as the temperature decreases to *T* = 345 K, only those alloys with *x*_Pd_ ≥ 0.99 displayed sufficient activity to estimate *n*_H2_. At all temperatures, when *P*_O2_^in^ = 0 Torr, *n*_H2_ is constant at a value between 0.5 and 1 and appears to display no composition dependence. On the other hand, when *P*_O2_^in^ = 15 Torr, *n*_H2_ at the same reaction temperatures is uniformly lower at high Pd content, with a value of ∼0. The change in *n*_H2_ depending on the presence of oxygen indicates a change in the reaction mechanism for ethylene hydrogenation on Pd versus on PdO_*x*_. Plots c–f also show that when *P*_O2_^in^ = 15 Torr, *n*_H2_ is highly sensitive to alloy composition: *n*_H2_ quickly transitions from ∼0 to the value of *n*_H2_ at *P*_O2_^in^ = 0 Torr when *x*_Pd_ is diluted with Ag by a few percent.

Figure 10 shows all of the values of *n*_H2_ that can be calculated using the data sets at *P*_O2_^in^ = 0 Torr (blue) and *P*_O2_^in^ = 15 Torr (red) versus *x*_Pd_. The plots were generated by finding the slopes of the best-fit line of log(ξ) versus log(*P*_H2_^in^) for the low conversion data at each catalyst composition (as in Figure S2). The error bars on *n*_H2_ in Figure 10 represent one standard deviation, as determined by the solver. The key point is to highlight the differences in *n*_H2_ between the data set where *P*_O2_^in^ = 0 Torr and the data set where *P*_O2_^in^ = 15 Torr. Even though at *P*_O2_^in^ = 0 Torr, *n*_H2_ can only be calculated for the most Pd-rich alloys, *n*_H2_ is constant at a positive value between 0.5 and 1 at all temperatures, showing virtually no composition dependence. On the other hand, when *P*_O2_^in^ = 15 Torr, *n*_H2_ ≈ 0 for the most Pd-rich alloys. The difference in *n*_H2_ for *P*_O2_^in^ = 0 Torr and *P*_O2_^in^ = 15 Torr at identical alloy compositions (including *x*_Pd_ = 1) indicates a difference in the reaction mechanism resulting from the changing nature of the catalyst surface, explained earlier as the simultaneous resurfacing and activation of Pd. Thus, the change in *n*_H2_ by O_2_ provides additional evidence for the restructuring of the catalyst surface proposed in Figure 8.

Figure 10 also reveals the sensitivity of *n*_H2_ to alloy composition when *P*_O2_^in^ = 15 Torr. It appears that alloys with a sufficiently high bulk fraction of Pd, *x*_Pd_ > 0.98 at *T* = 330 K decreasing to *x*_Pd_ > 0.94 at *T* = 375 K, all behave like a clean surface of PdO_*x*_ (i.e., *x*_Pd_ = 1 with *P*_O2_^in^ = 15 Torr), which has *n*_H2_ ≈ 0 for ethylene hydrogenation. This is clearly lower than the value of *n*_H2_ in the absence of O_2_. For these high *x*_Pd_ alloy compositions, we conclude that *P*_O2_^in^ = 15 Torr at the given reaction temperature was sufficient to resegregate Pd, leading to the formation of a top surface entirely composed of PdO_*x*_, as shown in Figure 8b. However, as *x*_Pd_ decreases, *n*_H2_ increases across a narrow composition range (Figure 10c through f) until it reaches values similar to those found on alloys with higher *x*_Pd_ when no O_2_ is present during the reaction. The sensitivity of *n*_H2_ to *x*_Pd_ underscores the effect of the bulk alloy composition on the overall surface structure and, consequently, on the reaction mechanism and catalytic activity for ethylene hydrogenation. Ultimately, this reveals the intricacy of the surface segregation phenomena at play, which involves the competition between the driving force for Pd resurfacing and activation caused by the presence of O_2_ and the driving force for the preservation of a Ag-capped surface that originally minimizes the surface free energy of the alloy. We observe that *n*_H2_ sensitivity to *x*_Pd_ appears to become slightly diminished at higher reaction temperatures, as indicated by the fact that *n*_H2_ remains constant between 0.6 < *x*_Pd_ < 0.9 when *T* = 405 K and *P*_O2_^in^ = 15 Torr (the red points in Figure 10a). The decreased sensitivity of *n*_H2_ to *x*_Pd_ at higher *T* may suggest that the driving force for Pd resegregation and activation by O_2_ becomes dominant when the temperature is increased since dilution with ∼30% Ag appears to have no effect on the reaction order. Regardless, it is evident from Figure 10 that *x*_Pd_, *T*, and *P*_O2_^in^ all impact *n*_H2_, which highlights the complexity of the interactions that determine a catalyst’s final structure and performance.

To briefly summarize our findings, by measuring the ethylene conversion, ξ(*x*), across Ag_*x*_Pd_1–*x*_ composition space, we have determined that *P*_O2_^in^ = 15 Torr in the feed activates alloys with as little as *x*_Pd_ = 0.6, enabling them to display measurable conversion of ethylene to ethane. This provides evidence to support the proposed rearrangement of AgPd catalyst surfaces that occurs in the presence of O_2_ (Figure 8). Analysis of *G*^‡^ and *n*_H2_ across composition space shows that the free energy of activation and the reaction order with respect to hydrogen are highly sensitive to relatively minor dilutions with Ag. When the bulk composition of Pd is sufficient to form a top surface composed entirely of PdO_*x*_ in the presence of O_2_, *G*^‡^ is at its minimum and the reaction order of ethylene hydrogenation with respect to hydrogen is *n*_H2_ ≈ 0, which matches the measurements of *G*^‡^ and *n*_H2_ at the point on the CSAF representing a clean PdO_*x*_ surface. Dilution of Pd with just a few percent of Ag causes a rapid increase in *n*_H2_ to positive values similar to those found for the mostly Ag-capped surface identified at *P*_O2_^in^ = 0 Torr. The value of *x*_Pd_ at which this transition occurs changes as a function of the reaction temperature due to the competition between the two opposing surface segregation tendencies at play, namely, for Ag to exist on the top surface due to its lower surface free energy and for Pd to exist on the top surface due to favorable interactions with O_2_. Thus, our high-throughput catalytic activity measurements of ethylene hydrogenation provide experimental evidence for surface segregation phenomena of the AgPd alloy system while highlighting the breadth of functional catalyst surfaces that can be present under different operating conditions.

## 5 Conclusions

This work presented a high-throughput investigation of the ethylene hydrogenation activity of a Ag_*x*_Pd_1–*x*_ CSAF. Two data sets were collected across a range of temperatures (*T* = 300 K → 405 K) and hydrogen pressures (*P*_H2_^in^ = 70 Torr → 690 Torr) with a constant inlet ethylene pressure of *P*_C2H4_^in^ = 25 Torr. In the data set with *P*_O2_^in^ = 0 Torr, only those alloys with a bulk composition of *x*_Pd_ ≥ 0.9 displayed any measurable conversion of ethylene to ethane. On the other hand, when *P*_O2_^in^ = 15 Torr, a significant increase in the conversion was observed for all previously active compositions and also for alloys with as little as *x*_Pd_ = 0.6 that were otherwise inactive. The stark difference in the ethylene hydrogenation activity is attributed to the favorable restructuring of Ag_*x*_Pd_1–*x*_ alloy surfaces in the presence of O_2_. First, the interaction of O_2_ with Pd creates an activated layer of PdO_*x*_, which was found to be a more efficient catalyst for the reaction than Pd itself. At the same time, the presence of O_2_ facilitates inverse surface segregation effects in the alloys, drawing Pd atoms to the top surface to replace the excess Ag that originally minimizes the surface free energy. We have estimated the free energy of activation, *G*^‡^, for ethylene hydrogenation on the AgPd catalysts, which decreases to its minimum value of ∼1.4 kJ/mol on clean PdO_*x*_ surfaces with *P*_O2_^in^ = 15 Torr and increases rapidly as both *x*_Pd_ and *P*_H2_^in^ decrease, especially when no O_2_ is present in the feed. We also estimated the reaction order for ethylene hydrogenation with respect to hydrogen, *n*_H2_, to show that *n*_H2_ changes from a positive value between 0.5 and 1 for metallic Pd binary alloys with mostly Ag-capped surfaces to *n*_H2_ ≈ 0 for PdO_*x*_ when *P*_O2_^in^ = 15 Torr. As *x*_Pd_ decreases below the critical value necessary to form a clean surface of PdO_*x*_, *n*_H2_ increases rapidly over a narrow composition range until it reaches positive values similar to those measured on AgPd alloys when *P*_O2_^in^ = 0 Torr. The *x*_*Pd*_ composition range at which this transition occurs changes as a function of the reaction temperature due to the competition between the driving forces for Pd and Ag segregation. By analyzing the changes in the catalytic performance of the Ag_*x*_Pd_1–*x*_ CSAF, we have helped to elucidate the behavior of this binary alloy system with unprecedented resolution in composition while also highlighting the importance of understanding adsorbate–catalyst interactions and changes to the catalyst surface under different operating conditions.

## Supporting Information Available

Calibration experiments showing a linear response between the mass spectrometer signal intensities at *m*/*z* = 29 and 30 amu and different emulated extents of ethylene conversion, ξ. Such a relationship permits linear interpolation of the 0 and 100% conversion reference channels for determining ξ inside the 100 microreactor channels; plots of log(ξ) versus log(*P*_H2_^in^) for a selected set of reaction conditions and alloy compositions to demonstrate how linear fitting was performed within a limited range of ethylene conversion values to obtain estimates of *n*_H2_(PDF)

## References

[ref1] Augustine R. L. Selective Heterogeneously Catalyzed Hydrogenations. Catal. Today 1997, 37 (4), 419–440. 10.1016/S0920-5861(97)00025-4.

[ref2] Nerozzi F. Heterogeneous Catalytic Hydrogenation. Platinum Met. Rev. 2012, 56 (4), 236–241. 10.1595/147106712X654187.

[ref3] Lin T.; Gong K.; Wang C.; An Y.; Wang X.; Qi X.; Li S.; Lu Y.; Zhong L.; Sun Y. Fischer–Tropsch Synthesis to Olefins: Catalytic Performance and Structure Evolution of Co_2_C-Based Catalysts under a CO_2_ Environment. ACS Catal. 2019, 9 (10), 9554–9567. 10.1021/acscatal.9b02513.

[ref4] Claus P. Selective Hydrogenation of α,β-Unsaturated Aldehydes and Other C=O and C=C Bonds Containing Compounds. Top. Catal. 1998, 5, 51–62. 10.1023/A:1019177330810.

[ref5] Zaera F.; Somorjai G. A. Hydrogenation of Ethylene over Platinum (111) Single-Crystal Surfaces. J. Am. Chem. Soc. 1984, 106 (8), 2288–2293. 10.1021/ja00320a013.

[ref6] Bos A. N. R.; Westerterp K. R. Mechanism and Kinetics of the Selective Hydrogenation of Ethyne and Ethene. Chem. Eng. Process. 1993, 32, 1–7. 10.1016/0255-2701(93)87001-B.

[ref7] Godbey D.; Zaera F.; Yeates R.; Somorjai G. A. Hydrogenation of Chemisorbed Ethylene on Clean, Hydrogen, and Ethylidyne Covered Platinum (111) Crystal Surfaces. Surf. Sci. 1986, 167 (1), 150–166. 10.1016/0039-6028(86)90791-0.

[ref8] Horiuti J.; Miyahara K. Mechanism of Catalyzed Hydrogenation of Ethylene in the Presence of Metallic Catalysts. Z. Phys. Chem. 1969, 64 (1–4), 36–40. 10.1524/zpch.1969.64.1_4.036.

[ref9] Salciccioli M.; Chen Y.; Vlachos D. G. Microkinetic Modeling and Reduced Rate Expressions of Ethylene Hydrogenation and Ethane Hydrogenolysis on Platinum. Ind. Eng. Chem. Res. 50, 28 40. 10.1021/ie100364a.

[ref10] Priyadarshini D.; Kondratyuk P.; Miller J. B.; Gellman A. J. Compact Tool for Deposition of Composition Spread Alloy Films. J. Vac. Sci. Technol., A 2012, 30 (1), 011503 10.1116/1.3664078.

[ref11] Fleutot B.; Miller J. B.; Gellman A. J. Apparatus for Deposition of Composition Spread Alloy Films: The Rotatable Shadow Mask. J. Vac. Sci. Technol., A 2012, 30, 061511 10.1116/1.4766194.

[ref12] Kondratyuk P.; Gumuslu G.; Shukla S.; Miller J. B.; Morreale B. D.; Gellman A. J. A Microreactor Array for Spatially Resolved Measurement of Catalytic Activity for High-Throughput Catalysis Science. J. Catal. 2013, 300, 55–62. 10.1016/j.jcat.2012.12.015.

[ref13] Binder A.; Seipenbusch M.; Muhler M.; Kasper G. Kinetics and Particle Size Effects in Ethene Hydrogenation over Supported Palladium Catalysts at Atmospheric Pressure. J. Catal. 2009, 268 (1), 150–155. 10.1016/j.jcat.2009.09.013.

[ref14] Sprunger P. T.; Plummer E. W. Interaction of Hydrogen with the Ag(110) Surface. Phys. Rev. B 1993, 48 (19), 14436 10.1103/PhysRevB.48.14436. 10007863

[ref15] Khan N. A.; Shaikhutdinov S.; Freund H. J. Acetylene and Ethylene Hydrogenation on Alumina Supported Pd-Ag Model Catalysts. Catal. Lett. 2006, 108, 159–164. 10.1007/s10562-006-0041-y.

[ref16] Zhang Q.; Li J.; Liu X.; Zhu Q. Synergetic Effect of Pd and Ag Dispersed on Al_2_O_3_ in the Selective Hydrogenation of Acetylene. Appl. Catal., A 2000, 197 (2), 221–228. 10.1016/S0926-860X(99)00463-9.

[ref17] Wachs I. E.; Madix R. J. The Oxidation of Methanol on a Silver (110) Catalyst. Surf. Sci. 1978, 76 (2), 531–558. 10.1016/0039-6028(78)90113-9.

[ref18] Pachulski A.; Schödel R.; Claus P. Performance and Regeneration Studies of Pd-Ag/Al_2_O_3_ Catalysts for the Selective Hydrogenation of Acetylene. Appl. Catal., A 2011, 400 (1–2), 14–24. 10.1016/j.apcata.2011.03.019.

[ref19] Ravanchi M. T.; Sahebdelfar S. Pd-Ag/Al_2_O_3_ Catalyst: Stages of Deactivation in Tail-End Acetylene Selective Hydrogenation. Appl. Catal., A 2016, 525, 197–203. 10.1016/j.apcata.2016.07.014.

[ref20] Pei G. X.; Liu X. Y.; Wang A.; Lee A. F.; Isaacs M. A.; Li L.; Pan X.; Yang X.; Wang X.; Tai Z.; Wilson K.; Zhang T. Ag Alloyed Pd Single-Atom Catalysts for Efficient Selective Hydrogenation of Acetylene to Ethylene in Excess Ethylene. ACS Catal. 2015, 5 (6), 3717–3725. 10.1021/acscatal.5b00700.

[ref21] Lamb R. N.; Ngamsom B.; Trimm D. L.; Gong B.; Silveston P. L.; Praserthdam P. Surface Characterisation of Pd-Ag/Al_2_O_3_ Catalysts for Acetylene Hydrogenation Using an Improved XPS Procedure. Appl. Catal., A 2004, 268 (1–2), 43–50. 10.1016/j.apcata.2004.03.041.

[ref22] Aich P.; Wei H.; Basan B.; Kropf A. J.; Schweitzer N. M.; Marshall C. L.; Miller J. T.; Meyer R. Single-Atom Alloy Pd-Ag Catalyst for Selective Hydrogenation of Acrolein. J. Phys. Chem. C 2015, 119 (32), 18140–18148. 10.1021/acs.jpcc.5b01357.

[ref23] Backx C.; de Groot C. P. M.; Biloen P. Electron Energy Loss Spectroscopy and its Applications. Appl. Surf. Sci. 1980, 6 (3–4), 256–272. 10.1016/0378-5963(80)90016-1.

[ref24] Barteau M. A.; Madix R. J. Low-Pressure Oxidation Mechanism and Reactivity of Propylene on Ag(110) and Relation to Gas-Phase Acidity. J. Am. Chem. Soc. 1983, 105 (3), 344–349. 10.1021/ja00341a008.

[ref25] Ayre C. R.; Madix R. J. π, π-Allyl, and Trimethylenemethane Complexes Derived from Isobutylene Adsorption on Oxygen-Activated Ag(110). Surf. Sci. 1992, 262 (1–2), 51–67. 10.1016/0039-6028(92)90459-J.

[ref26] Williams F. J.; Bird D. P. C.; Charles E.; Sykes H.; Santra A. K.; Lambert R. M. Molecular Conformation of Styrene on Ag(100): Relevance to an Understanding of the Catalytic Epoxidation of Terminal Alkenes. J. Phys. Chem. B 2003, 107 (16), 3824–3828. 10.1021/jp027422a.

[ref27] Klust A.; Madix R. J. Partial Oxidation of Higher Olefins on Ag(1 1 1): Conversion of Styrene to Styrene Oxide, Benzene, and Benzoic Acid. Surf. Sci. 2006, 600 (23), 5025–5040. 10.1016/j.susc.2006.08.049.

[ref28] Barteau M. A.; Madix R. J. Acetylenic Complex Formation and Displacement via Acid-Base Reactions on Ag(110). Surf. Sci. 1982, 115 (2), 355–381. 10.1016/0039-6028(82)90415-0.

[ref29] Stuve E. M.; Madix R. J.; Sexton B. A. Characterization of the Adsorption and Reaction of Acetylene on Clean and Oxygen Covered Ag(110) by EELS. Surf. Sci. 1982, 123 (2–3), 491–504. 10.1016/0039-6028(82)90342-9.

[ref30] Barteau M. A.; Bowker M.; Madix R. J. Acid-Base Reactions on Solid Surfaces: The Reactions of HCOOH, H_2_CO, and HCOOCH_3_ with Oxygen on Ag(110). Surf. Sci. 1980, 94 (2–3), 303–322. 10.1016/0039-6028(80)90009-6.

[ref31] Zhou L.; Madix R. J. Oxidation of Styrene and Phenylacetaldehyde on Ag(111): Evidence for Transformation of Surface Oxametallacycle. J. Phys. Chem. C 2008, 112 (12), 4725–4734. 10.1021/jp7119558.

[ref32] Brainard R. L.; Madix R. J. Oxidation of Tert-Butyl Alcohol to Isobutylene Oxide on a Ag(110) Surface: The Role of Unactivated C-H Bonds in Product Selectivity. J. Am. Chem. Soc. 1989, 111 (11), 3826–3835. 10.1021/ja00193a012.

[ref33] Ayre C. R.; Madix R. J. The Adsorption and Reaction of 1,2-Propanediol on Ag(110) under Oxygen Lean Conditions. Surf. Sci. 1994, 303 (3), 279–296. 10.1016/0039-6028(94)90776-5.

[ref34] Zhou X. L.; White J. M.; Koel B. E. Chemisorption of Atomic Hydrogen on Clean and Cl-Covered Ag(111). Surf. Sci. 1989, 218 (1), 201–210. 10.1016/0039-6028(89)90627-4.

[ref35] Lee G.; Sprunger P. T.; Okada M.; Poker D. B.; Zehner D. M.; Plummer E. W. Chemisorption of Hydrogen on the Ag(111) Surface. J. Vac. Sci. Technol., A 1994, 12 (4), 2119–2123. 10.1116/1.579147.

[ref36] Conrad H.; Ertl G.; Latta E. E. Adsorption of Hydrogen on Palladium Single Crystal Surfaces. Surf. Sci. 1974, 41 (2), 435–446. 10.1016/0039-6028(74)90060-0.

[ref37] Ferrin P.; Kandoi S.; Nilekar A. U.; Mavrikakis M. Hydrogen Adsorption, Absorption and Diffusion on and in Transition Metal Surfaces: A DFT Study. Surf. Sci. 2012, 606, 679–689. 10.1016/j.susc.2011.12.017.

[ref38] Herron J. A.; Tonelli S.; Mavrikakis M. Atomic and Molecular Adsorption on Pd(111). Surf. Sci. 2012, 606, 1670–1679. 10.1016/j.susc.2012.07.003.

[ref39] Golio N.; Sen I.; Guo Z.; Railkar R.; Gellman A. J. Kinetic Parameter Estimation for Catalytic H_2_–D_2_ Exchange on Pd. Catal. Lett. 2023, 153, 1–18. 10.1007/s10562-022-03961-0.

[ref40] Skriver H. L.; Rosengaard N. M. Surface Energy and Work Function of Elemental Metals. Phys. Rev. B 1992, 46 (11), 7157 10.1103/PhysRevB.46.7157. 10002423

[ref41] de Boer F R.; Boom R.; Matterns W C. M.; Miedema A. R. Cohesion in Metals: Transition Metal Alloys (Cohesion and Structure); 1988.

[ref42] Wouda P. T.; Schmid M.; Nieuwenhuys B. E.; Varga P. STM Study of the (111) and (100) Surfaces of PdAg. Surf. Sci. 1998, 417 (2–3), 292–300. 10.1016/S0039-6028(98)00673-6.

[ref43] Wouda P. T.; Schmid M.; Nieuwenhuys B. E.; Varga P. Adsorbate Migration on PdAg(111). Surf. Sci. 1999, 423 (1), 1229–1235. 10.1016/S0039-6028(98)00937-6.

[ref44] Walle L. E.; Grönbeck H.; Fernandes V. R.; Blomberg S.; Farstad M. H.; Schulte K.; Gustafson J.; Andersen J. N.; Lundgren E.; Borg A. Surface Composition of Clean and Oxidized Pd_75_Ag_25_(100) from Photoelectron Spectroscopy and Density Functional Theory Calculations. Surf. Sci. 2012, 606 (23–24), 1777–1782. 10.1016/j.susc.2012.07.006.

[ref45] Vignola E.; Steinmann S. N.; Vandegehuchte B. D.; Curulla D.; Sautet P. C_2_H_2_-Induced Surface Restructuring of Pd-Ag Catalysts: Insights from Theoretical Modeling. J. Phys. Chem. C 2016, 120 (46), 26320–26327. 10.1021/acs.jpcc.6b08524.

[ref46] Vignola E.; Steinmann S. N.; Le Mapihan K.; Vandegehuchte B. D.; Curulla D.; Sautet P. Acetylene Adsorption on Pd-Ag Alloys: Evidence for Limited Island Formation and Strong Reverse Segregation from Monte Carlo Simulations. J. Phys. Chem. C 2018, 122 (27), 15456–15463. 10.1021/acs.jpcc.8b04108.

[ref47] Van Spronsen M. A.; Daunmu K.; O’Connor C. R.; Egle T.; Kersell H.; Oliver-Meseguer J.; Salmeron M. B.; Madix R. J.; Sautet P.; Friend C. M. Dynamics of Surface Alloys: Rearrangement of Pd/Ag(111) Induced by CO and O_2_. J. Phys. Chem. C 2019, 123 (13), 8312–8323. 10.1021/acs.jpcc.8b08849.

[ref48] Kumar D.; Han Y. F.; Goodman D. W. Ethylene Combustion on Unsupported and Supported Pd: A Comparative Study. Top. Catal. 2007, 46 (1–2), 169–174. 10.1007/s11244-007-0327-3.

[ref49] Wei T.; Wang J.; Goodman D. W. Characterization and Chemical Properties of Pd-Au Alloy Surfaces. J. Phys. Chem. C 2007, 111, 8781–8788. 10.1021/jp067177l.

[ref50] Priyadarshini D.; Kondratyuk P.; Picard Y. N.; Morreale B. D.; Gellman A. J.; Miller J. B. High-Throughput Characterization of Surface Segregation in Cu_x_Pd_1-x_ Alloys. J. Phys. Chem. C 2011, 115, 10155–10163. 10.1021/jp201793d.

[ref51] He J.-W.; Shea W.-L.; Jiang X.; Goodman D. W. Surface Chemistry of Monolayer Metallic Films on Re(0001) and Mo(110). J. Vac. Sci. Technol., A 1990, 8, 2435–2444. 10.1116/1.576711.

[ref52] Park C.; Bauer E.; Poppa H. Growth and Alloying of Pd Films on Mo(110) Surfaces. Surf. Sci. 1985, 154, 371–393. 10.1016/0039-6028(85)90040-8.

[ref53] Boes J. R.; Kondratyuk P.; Yin C.; Miller J. B.; Gellman A. J.; Kitchin J. R. Core Level Shifts in Cu-Pd Alloys as a Function of Bulk Composition and Structure. Surf. Sci. 2015, 640, 127–132. 10.1016/j.susc.2015.02.011.

[ref54] Goldstein J. I.; Newbury D. E.; Michael J. R.; Ritchie N. W. M. M.; Scott J. H. J.; Joy D. C. Scanning Electron Microscopy and X-Ray Microanalysis; Springer, 2018.

[ref55] Álvarez-Galván M. C.; De La Peña O’Shea V. A.; Fierro J. L. G.; Arias P. L. Alumina-Supported Manganese- and Manganese-Palladium Oxide Catalysts for VOCs Combustion. Catal. Commun. 2003, 4 (5), 223–228. 10.1016/S1566-7367(03)00037-2.

[ref56] Chlebda D. K.; Jędrzejczyk R. J.; Jodłowski P. J.; Łojewska J. Surface Structure of Cobalt, Palladium, and Mixed Oxide-Based Catalysts and Their Activity in Methane Combustion Studied by Means of Micro-Raman Spectroscopy. J. Raman Spectrosc. 2017, 48 (12), 1871–1880. 10.1002/jrs.5261.

[ref57] Thampi K. R.; Kiwi J.; Grätzel M. Oxidative Dimerisation of Methane on Supported Palladium Oxide Catalysts. Catal. Lett. 1990, 4 (1), 49–55. 10.1007/BF00764870.

[ref58] Wang K.; Li W.; Huang J.; Huang J.; Zhan G.; Li Q. Enhanced Active Site Extraction from Perovskite LaCoO_3_ Using Encapsulated PdO for Efficient CO_2_ Methanation. J. Energy Chem. 2020, 53, 9–19. 10.1016/j.jechem.2020.05.027.

[ref59] Rout D. R.; Senapati P.; Sutar H.; Sau D. C.; Murmu R. Graphene Oxide (GO) Supported Palladium (Pd) Nanocomposites for Enhanced Hydrogenation. Graphene 2019, 08 (03), 33–51. 10.4236/graphene.2019.83003.

[ref60] Tew M. W.; Janousch M.; Huthwelker T.; Van Bokhoven J. A. The Roles of Carbide and Hydride in Oxide-Supported Palladium Nanoparticles for Alkyne Hydrogenation. J. Catal. 2011, 283 (1), 45–54. 10.1016/j.jcat.2011.06.025.

[ref61] Aliahmadi M.; Davoudi M.; Kharat A. N. Selective Hydrogenation of Phenol to Cyclohexanone Catalyzed by Palladium Nanoparticles Supported on Alumina/Lanthanide Oxides. React. Kinet., Mech. Catal. 2020, 131 (2), 819–828. 10.1007/s11144-020-01900-x.

[ref62] Fernandes V. R.; Van den Bossche M.; Knudsen J.; Farstad M. H.; Gustafson J.; Venvik H. J.; Grönbeck H.; Borg A. Reversed Hysteresis during CO Oxidation over Pd_75_Ag_25_(100). ACS Catal. 2016, 6 (7), 4154–4161. 10.1021/acscatal.6b00658.

[ref63] Svenum I. H.; Herron J. A.; Mavrikakis M.; Venvik H. J. Adsorbate-Induced Segregation in a PdAg Membrane Model System: Pd_3_Ag(111). Catal. Today 2012, 193, 111–119. 10.1016/j.cattod.2012.01.007.

[ref64] Løvvik O. M.; Opalka S. M. Reversed Surface Segregation in Palladium-Silver Alloys Due to Hydrogen Adsorption. Surf. Sci. 2008, 602 (17), 2840–2844. 10.1016/j.susc.2008.07.016.

[ref65] Gedara B. S. A.; Muir M.; Islam A.; Liu D.; Trenary M. Room Temperature Migration of Ag Atoms to Cover Pd Islands on Ag(111). J. Phys. Chem. C 2021, 125 (50), 27828–27836. 10.1021/acs.jpcc.1c08736.

[ref66] Kitchin J. R.; Reuter K.; Scheffler M. Alloy Surface Segregation in Reactive Environments: First-Principles Atomistic Thermodynamics Study of Ag_3_Pd(111) in Oxygen Atmospheres. Phys. Rev. B 2008, 77 (7), 075437 10.1103/PhysRevB.77.075437.

[ref67] O’connor C. R.; Van Spronsen M. A.; Egle T.; Xu F.; Kersell H. R.; Oliver-Meseguer J.; Karatok M.; Salmeron M.; Madix R. J.; Friend C. M. Hydrogen Migration at Restructuring Palladium-Silver Oxide Boundaries Dramatically Enhances Reduction Rate of Silver Oxide. Nat. Commun. 11, 1844. 10.1038/s41467-020-15536-x. PMC7160204 32296065

