# Supplementary material for: Activation by O2 of AgxPd1–x Alloy Catalysts for Ethylene Hydrogenation
Source: ACS Catal. 2023 Oct 28;13(22):14548–61. doi: 10.1021/acscatal.3c03253 (PMC10660651; doi:10.1021/acscatal.3c03253)
Supplement: Supplementary file 1 — cs3c03253_si_001.pdf [file cs3c03253_si_001.pdf]

Supporting Information

Activation by O_2 of Ag_xPd_{1-x} Alloy Catalysts for Ethylene Hydrogenation

Nicholas Golio[1] and Andrew J Gellman[1,2*]
[1]Department of Chemical Engineering
[2]W.E. Scott Institute for Energy Innovation
Carnegie Mellon University
5000 Forbes Ave., Pittsburgh, PA 15213, United States

*Corresponding author: gellman@cmu.edu, 412-268-3848

1. Linear interpolation of the mass spectrometer signal

Calibration experiments were performed to investigate whether linear interpolation of the mass spectrometer signal is appropriate for ethylene hydrogenation. In the 0% conversion reference channel, we flowed different gas mixtures of C_2H_4, C_2H_6, and Ar corresponding to different extents of reaction, ξ, to determine which signals increase (or decrease) linearly with respect to conversion. For example, a mixture containing P_{C2H4}^{in} = 25 Torr and P_{C2H6}^{in} = 0 corresponds to ξ = 0, while a mixture containing P_{C2H4}^{in} = 20 Torr and P_{C2H6}^{in} = 5 Torr corresponds to ξ = 0.2. Figure S1 shows the results of the calibration experiments for the signal intensities at *m/z* = 29 and 30 amu, which are the most likely candidates for the interpolation from Figure 1.

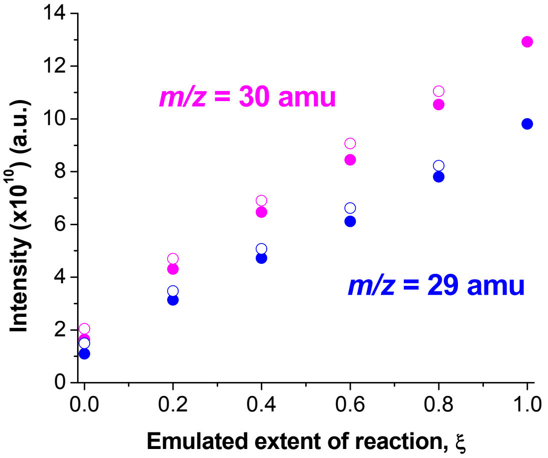

Figure S1. Mass spectrometer intensities at *m/z* = 29 and 30 amu in the 0% conversion reference channel versus ethylene/ethane ratios emulating conversion, ξ. The conversion was controlled by flowing the corresponding C_2H_4 to C_2H_6 ratio totaling 25 Torr in Ar (i.e. ξ = 0.2 corresponds to P_{C2H4}^{in} = 20 Torr + P_{C2H6}^{in} = 5 Torr). The experiment was performed by emulating the conversion sequentially from $\xi = 0 \rightarrow 1 \rightarrow 0$ and allowing the signals to reach steady-state before measuring their intensities. The filled symbols were obtained while increasing $\xi = 0 \rightarrow 1$ and the open symbols were obtained while decreasing $\xi = 1 \rightarrow 0$. Since all of the open symbols lie above the filled symbols, there is minimal yet uniform drift in the signals with time. The intensities at *m/z* = 29 and 30 amu are linear with respect to ξ, and as such, those mass spectrometer signals were used to quantify the ethylene conversion in the microreactor channels.

The filled symbols in Figure S1 correspond to incrementally changing the composition of the gas mixture by increasing the ethane/ethylene ratio to emulate $\xi = 0 \rightarrow 1$. The open symbols show the data obtained while decreasing $\xi = 1 \rightarrow 0$. Measuring the signal intensities emulating both increasing and decreasing ξ ensured that the observed trends were reproducible, and it also allowed us to quantify the drift of the mass spectrometer signal over the course of the experiment. Figure S1 shows that the signal intensities at *m/z* = 29 and 30 amu increase linearly with respect to ξ and that they also experience minimal drift. Given these considerations, linear interpolation of the signals at *m/z* = 29 and 30 amu was used to quantify the ethylene conversion in the microreactor channels.

2. Linear fitting of log (ξ) versus log (P_{H2}^{in}) to estimate n_{H2}

Figure S2 shows plots of log (ξ) versus log (P_{H2}^{in}) for the 20 most Pd-rich alloys ranging from $x_{Pd} = 1 \rightarrow 0.86$ under conditions where P_{O2}^{in} = 15 Torr and T = 375 K → 330 K. Only those points with low conversion, defined as $\xi < 0.3$ (log (ξ) < -0.52), were used to estimate the reaction order with respect to hydrogen, n_{H2}, in Figure 10. Note that all points with $\xi < 0.02$ (log (ξ) < -1.7) were also excluded from the analysis due to their conversion falling below the noise level. From the plots in Figure S2, it is clear that a line describes the relationship between log (ξ) and log (P_{H2}^{in}) and, therefore, the slope of the best fit line yields n_{H2}. The datapoints fitted by a line of the same color in Figure S2 show the experimental conditions where estimates of n_{H2} were made, while all other datapoints were excluded. Note that although it appears as though some of the low temperature data in Figure S2 are slightly curved, a linear fit was used for all datasets due to the number of points used for the fitting and the expectation that n_{H2} should not change with

P_{H2}^{in} on Pd surfaces. The slopes of the best fit lines estimate the average value of n_{H2} over an order of magnitude change in P_{H2}^{in}.

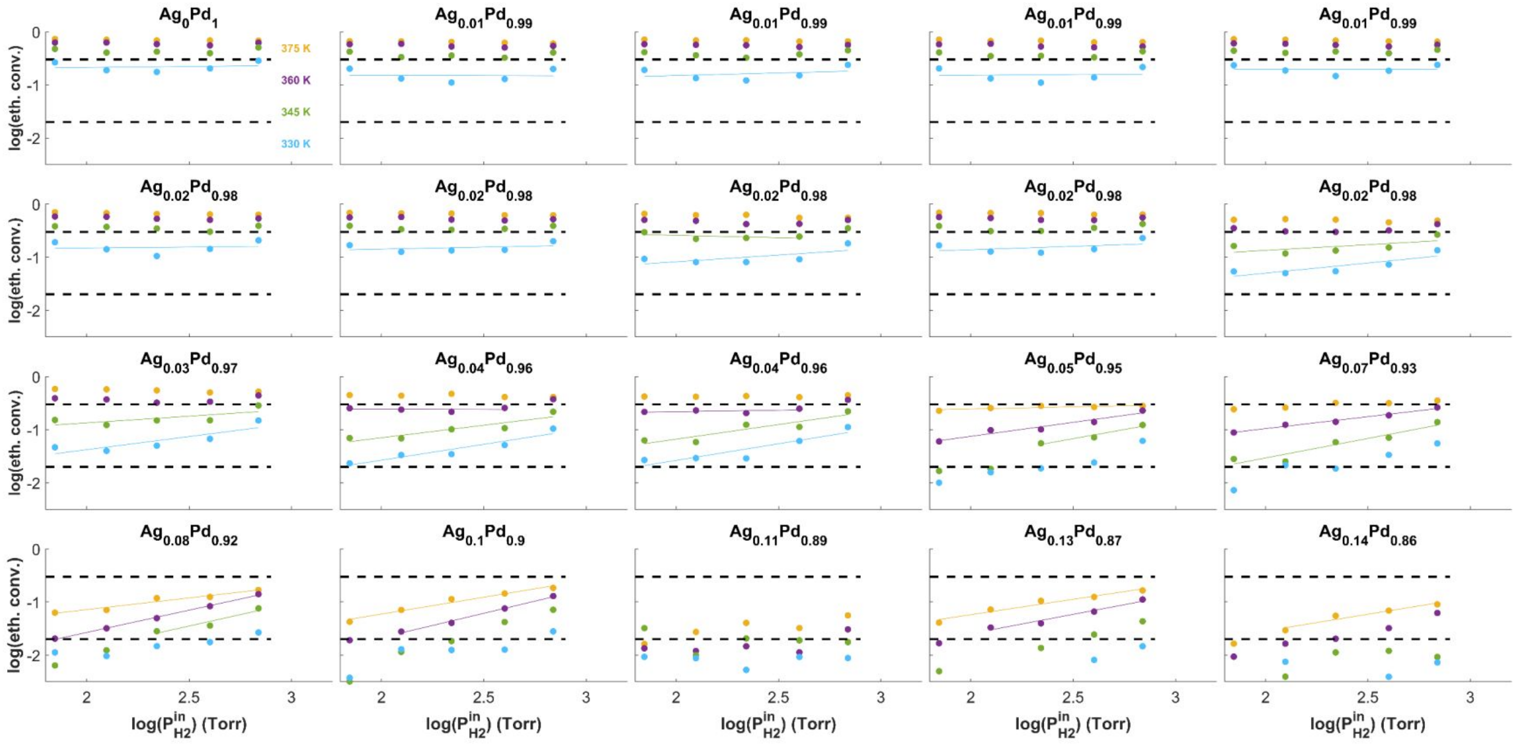

Figure S2. Logarithm of ethylene conversion, log (ξ), versus logarithm of P_{H2}^{in}, log (P_{H2}^{in}), for Ag_xPd_{1-x} alloys with $0.86 \leq x_{Pd} \leq 1$ when P_{O2}^{in} = 15 Torr and T = 375 K → 330 K. The low conversion data (i.e. $\xi < 0.3$) was used to fit a line describing the relationship between log (ξ) and log (P_{H2}^{in}) to estimate the reaction order with respect to hydrogen, n_{H2}. Only those datapoints fitted by a line of the same color were used to obtain estimates for n_{H2}, while all others were excluded due to the conversion being too high ($\xi > 0.3$) or below the level of noise ($\xi < 0.02$). The dotted black lines frame the range of values for log (ξ) that were used to obtain estimates of n_{H2}. Note that despite the apparent curvature on some plots at low T, a linear fit was selected for all datasets due to the number of points used for the fitting and the fact that the reaction order with respect to hydrogen is not expected to change with P_{H2}^{in} on Pd surfaces. In this way, the slope of the linear fit estimates the average n_{H2} across an order of magnitude change in P_{H2}^{in}. Linear fits were applied across both datasets with P_{O2}^{in} = 0 Torr and P_{O2}^{in} = 15 Torr to generate the plots seen in Figure 10.
